# Supplementary figures and images for: De novo Assembly of a 40 Mb Eukaryotic Genome from Short Sequence Reads: Sordaria macrospora, a Model Organism for Fungal Morphogenesis
Source: PLoS Genet. 2010 Apr 8;6(4):e1000891. doi: 10.1371/journal.pgen.1000891 (PMC2851567; doi:10.1371/journal.pgen.1000891)

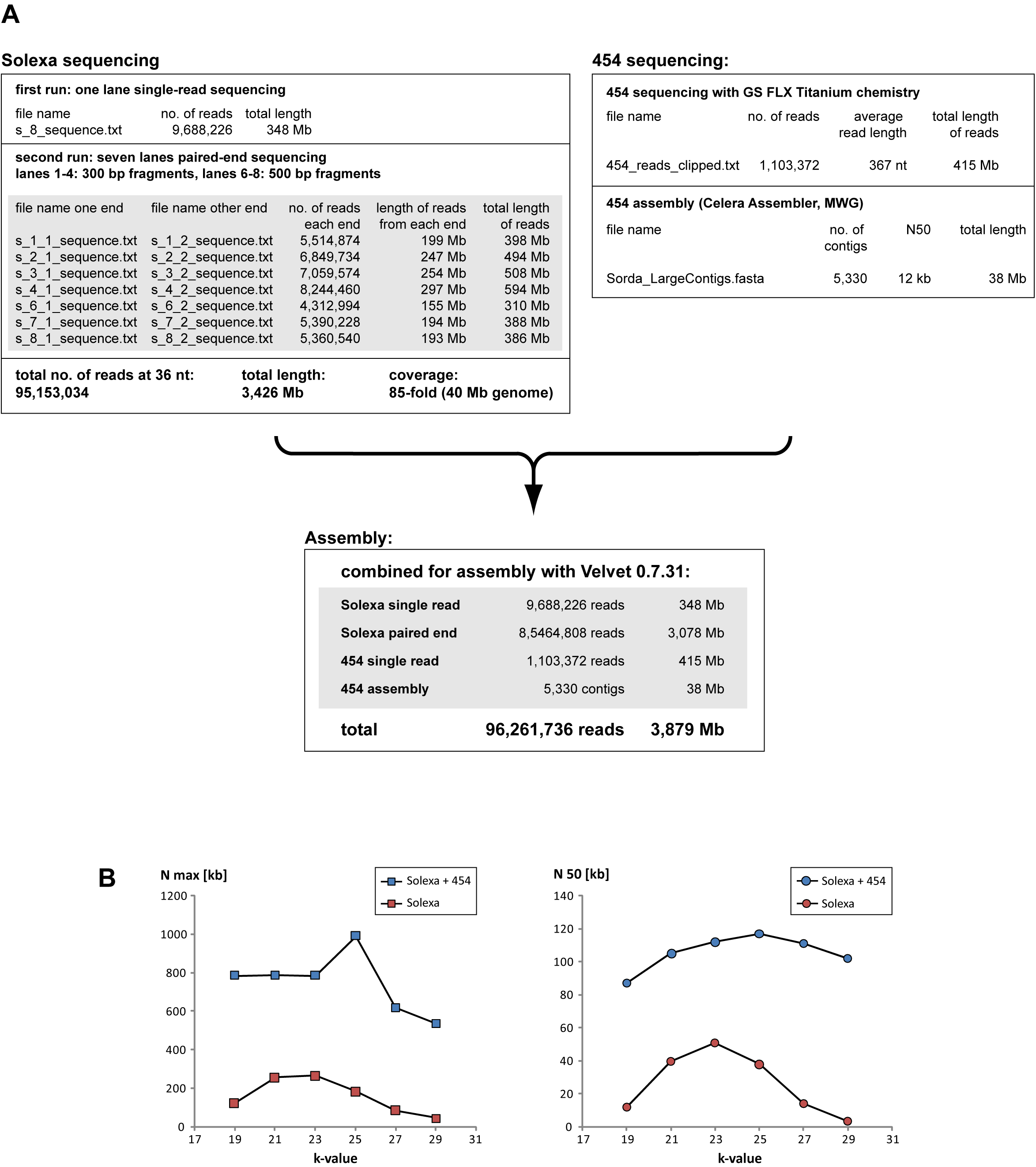

Supplement: Figure S1 — Next-generation sequencing of the S. macrospora genome. (A) Summary of the Illumina/Solexa and 454 sequences that were obtained. (B) Maximum contig lengths (N max) and N50 values for assemblies with different k-values (hash length in Velvet) for the Illumina/Solexa data alone or in combination with the 454 data. The combined assembly with the highest N max and N50 value (k = 25) was used for further analyses. (0.40 MB TIF) [file pgen.1000891.s001.tif]

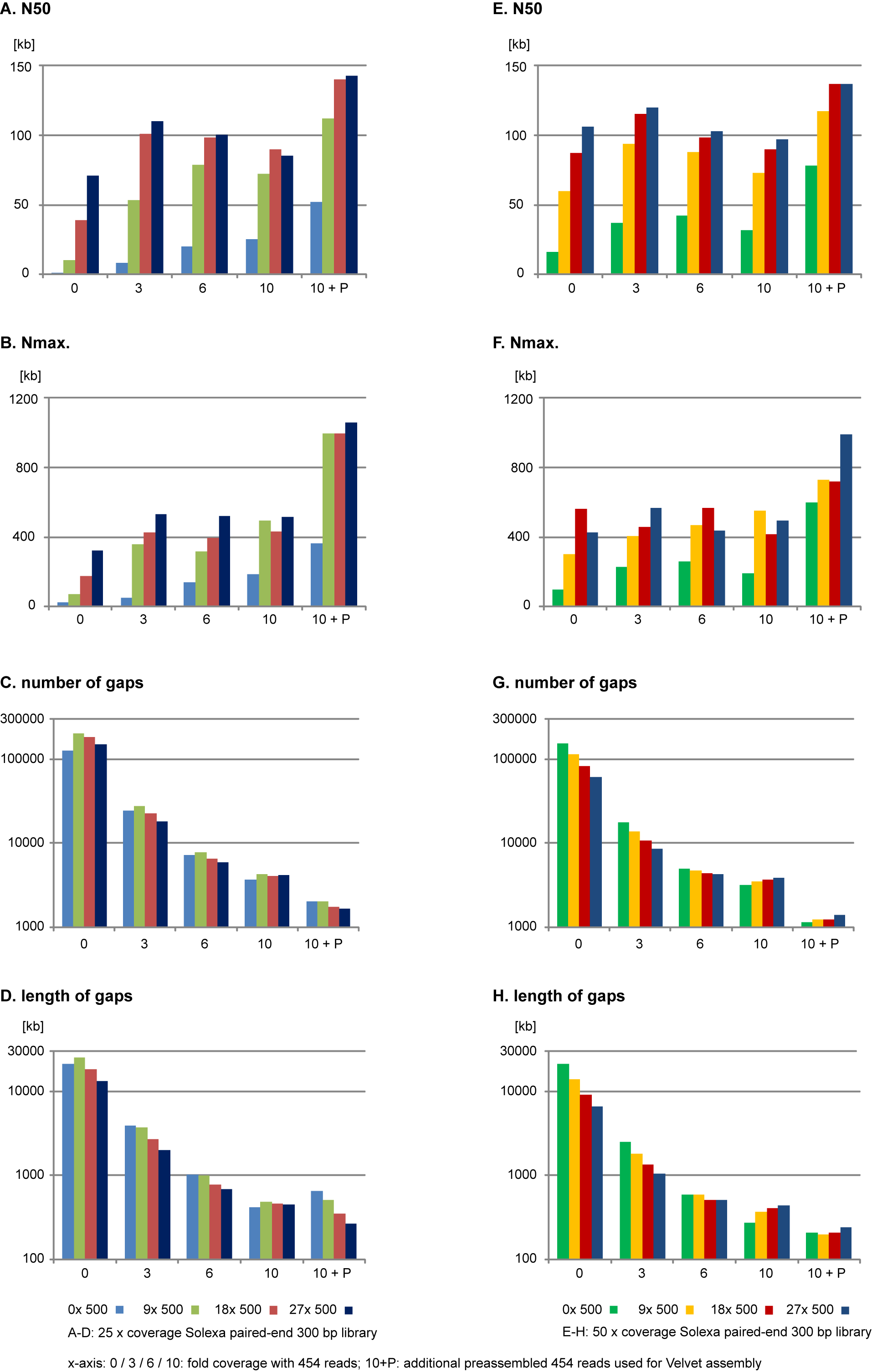

Supplement: Figure S2 — Assemblies with different coverage levels of short reads. Assemblies were done with Velvet 0.7.56 with k = 25. N50, maximum contig length (Nmax), the number of gaps that Velvet introduced within contigs and the total length of gaps within the assembly are given for different combinations of 454 coverage (x-axis), coverage from a 300 bp Solexa paired-end library (25× coverage in (A–D), 50× coverage in (E–H), respectively), and coverage from a 500 bp Solexa paired-end library (0×, 9×, 18×, and 27× coverage as color-coded in the different panels). Addition of 454 reads has the most drastic effect on the number and length of gaps (note the logarithmic y-axis for these panels) whereas addition of paired-end reads influences mostly N50 and Nmax. A table with assembly information for additional coverage combinations can be found in Table S1. (1.07 MB TIF) [file pgen.1000891.s002.tif]

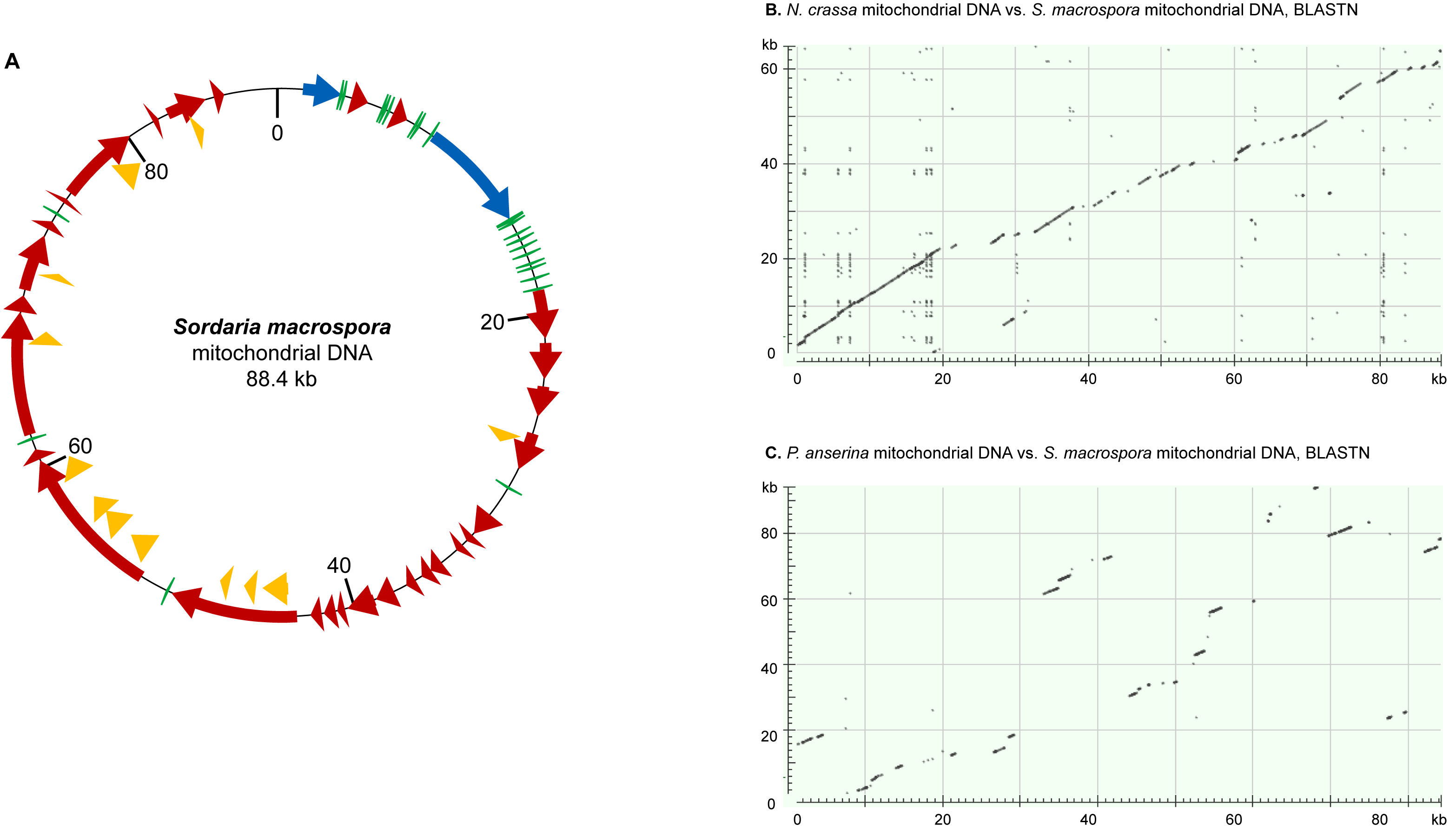

Supplement: Figure S3 — The mitochondrial genome of S. macrospora. (A) Schematic map of the mitochondrial genome. Size 88,423 bp, scale in kb indicated on the inner circle. Blue: ribosomal RNAs, green: tRNAs, red: protein coding genes, orange on the inner circle: open reading frames within introns of protein coding genes. Note that all predicted genes are encoded on the same strand. (B, C) Comparative analysis of the mitochondrial DNA of S. macrospora with N. crassa (B) and P. aserina (C). Dot plots of BLASTN analysis that was done at http://blast.ncbi.nlm.nih.gov/Blast.cgi with an e-value cutoff of 10−20. (0.35 MB TIF) [file pgen.1000891.s003.tif]

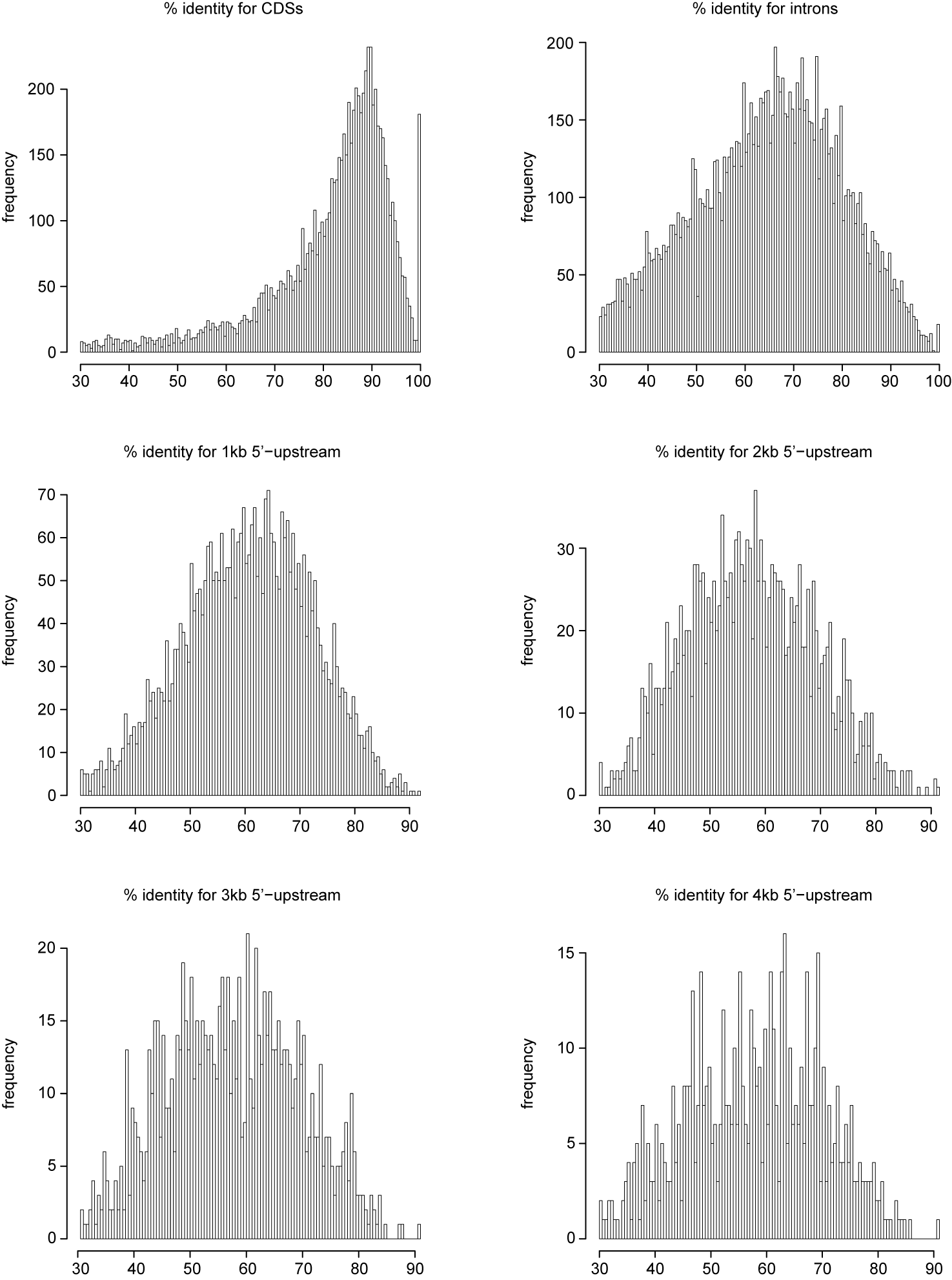

Supplement: Figure S4 — Histograms of % pairwise identity between S. macrospora and N. crassa for different genomic regions. CDSs, introns, and regions upstream of CDSs (in 1 kb steps ranging from 1 to 4 kb) were used for comparison. Only those upstream regions were used that do not overlap with a protein coding region. Each region was used only once even if it is upstream of two divergently transcribed genes to avoid double-counting. Detailed information on the comparisons can be found in Table S5. (0.65 MB TIF) [file pgen.1000891.s004.tif]

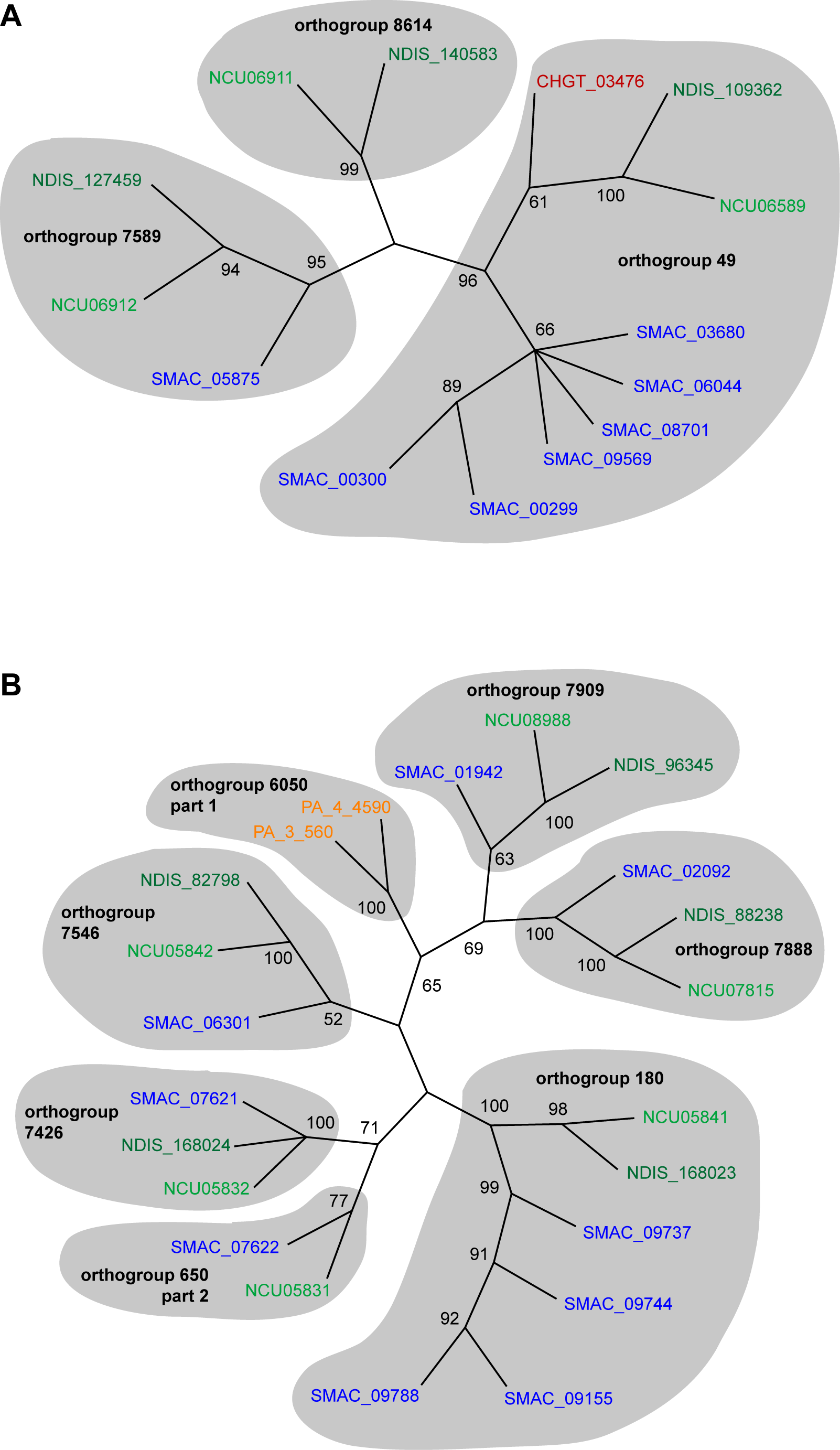

Supplement: Figure S5 — Phylogenetic analysis of orthogroups 49 (A) and 180 (B) and related orthogroups. With the exception of orthogroup 6050, which is split in two parts, all orthogroups that were found by OrthoMCL are supported by phylogenetic analysis. Most orthogroups contain one member each in S. macrospora, N. crassa, and N. discreta, but orthogroups 49 and 180 contain six and four members, respectively, from S. macrospora. Thus, both the OrthoMCL analysis as well as the phylogenetic trees constructed with maximum parsimony support the hypothesis that orthogroups 49 and 180 are part of larger gene families, but that in these branches of the gene families, recent gene duplication events occured specifically in S. macrospora. Numbers at branches indicate bootstrap support (10,000 bootstrap replications) in % for maximum parsimony trees. SM: S. macrospora, NC: N. crassa, ND: N. discreta, CHG: C. globosum, PA: P. anserina. (0.72 MB TIF) [file pgen.1000891.s005.tif]

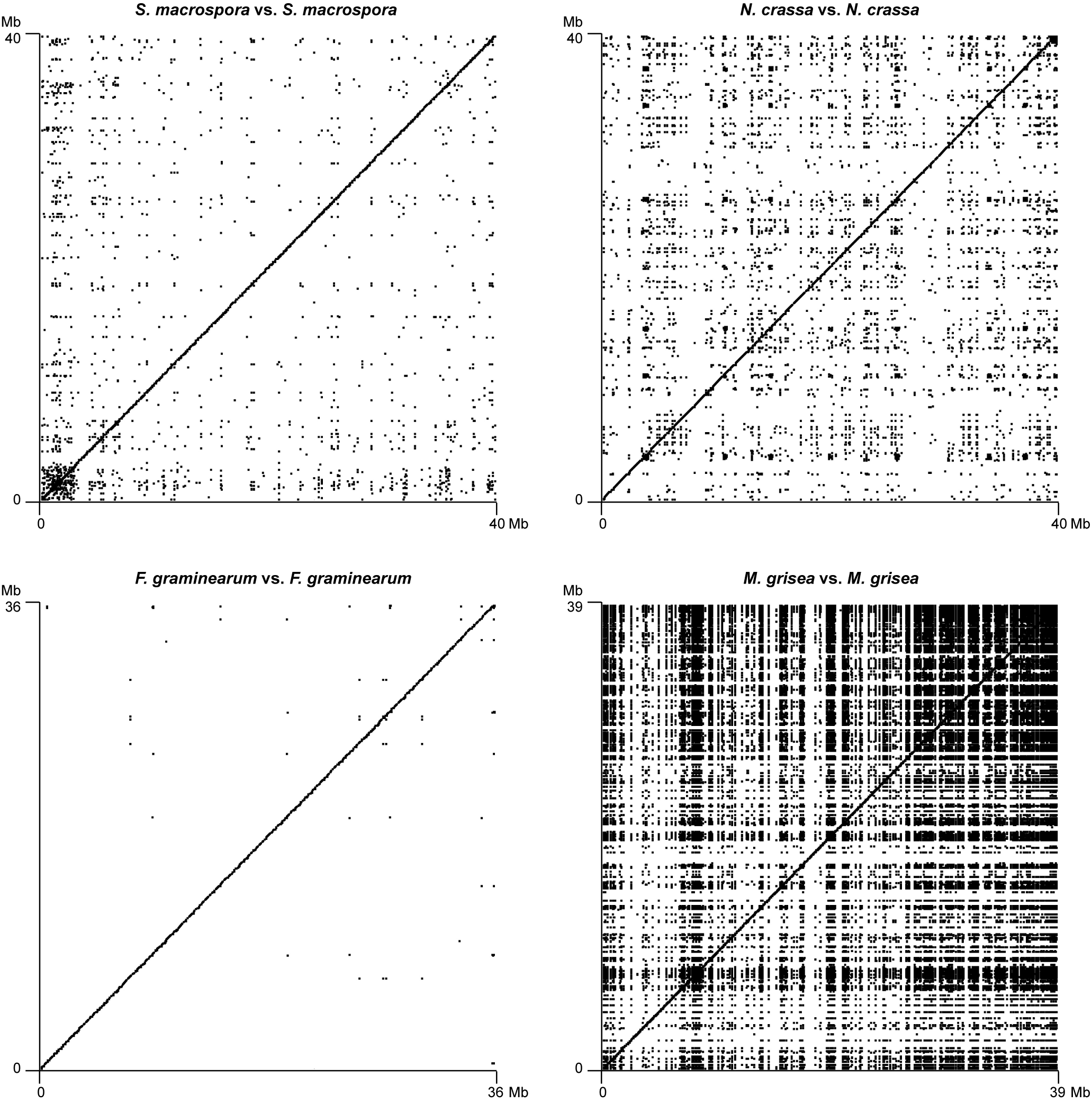

Supplement: Figure S6 — Regions of high similarity within four fungal genomes. Each genome sequence was compared to itself with BLASTN with e-value <10−150. Dot plot visualization was done with Combo (Engels et al. 2006, Bioinformatics 22: 1782–1783). The M. grisea genome contains a high amount of repeated DNA (Dean et al. 2005, Nature 434 :980–986), and this is reflected in this comparison. The genomes of N. crassa (Galagan et al. 2003, Nature 422: 859–868) and F. graminearum (Cuomo et al. 2007, Science 317: 1400–1402) contain only few repeat regions. The intragenomic similarities within the S. macrospora genome range between those for N. crassa and F. graminearum. (0.87 MB TIF) [file pgen.1000891.s006.tif]

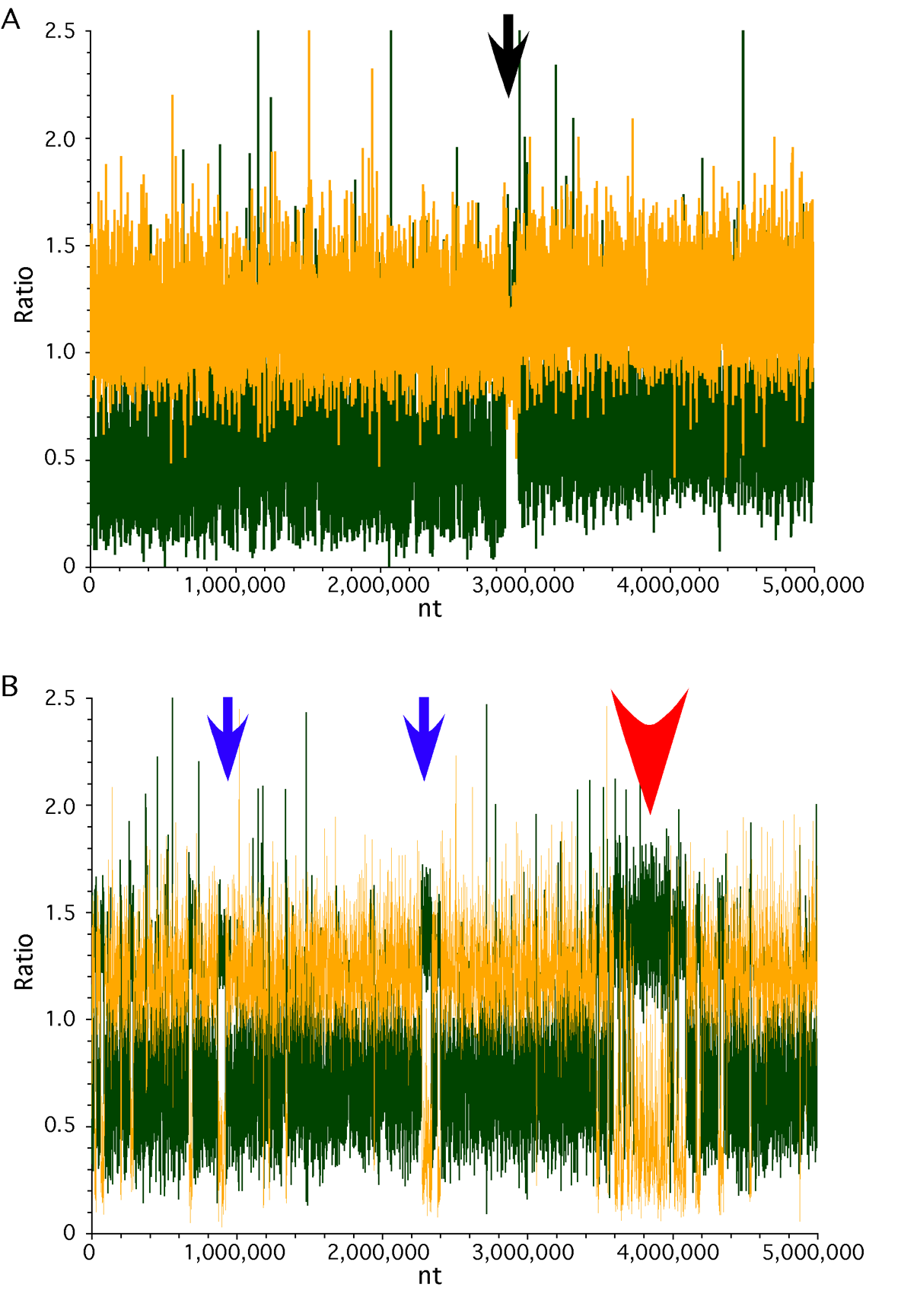

Supplement: Figure S7 — Comparison of RIP indices in the S.macrospora and N. crassa genomes. (A) No evidence for large regions with RIP in the S. macrospora genome. The substrate ([CA+TG]/[AC+GT]; orange) and product (TpA/ApT; dark green) RIP indices were calculated for all unscaffolded contigs (nt 1 to 2,865,981), the mtDNA (nt 2,865,982 to 2,954,404) and random scaffolds (nt 2,954,405 to 5,000,000). The patterns for the remainder of the S. macrospora genome look similar to those shown here for the random scaffolds. We predicted that the unscaffolded contigs would be AT-rich and would show hallmarks of RIP. Instead, these contigs are GC-rich and show now evidence for RIP by this assay. The mtDNA (black arrow) has balanced AT and GC content, more resembling bacterial DNA and thus has a different pattern than S. macrospora nuclear DNA. (B) Evidence for RIP in N. crassa Linkage Group I. The first 5 Mb of LGI of N. crassa were analyzed as above. High values for the product RIP index (green), coupled with low values for the substrate RIP index (orange) reveal dispersed (blue arrows) and centromeric (red arrowhead) regions that have been subjected to RIP. (0.57 MB TIF) [file pgen.1000891.s007.tif]

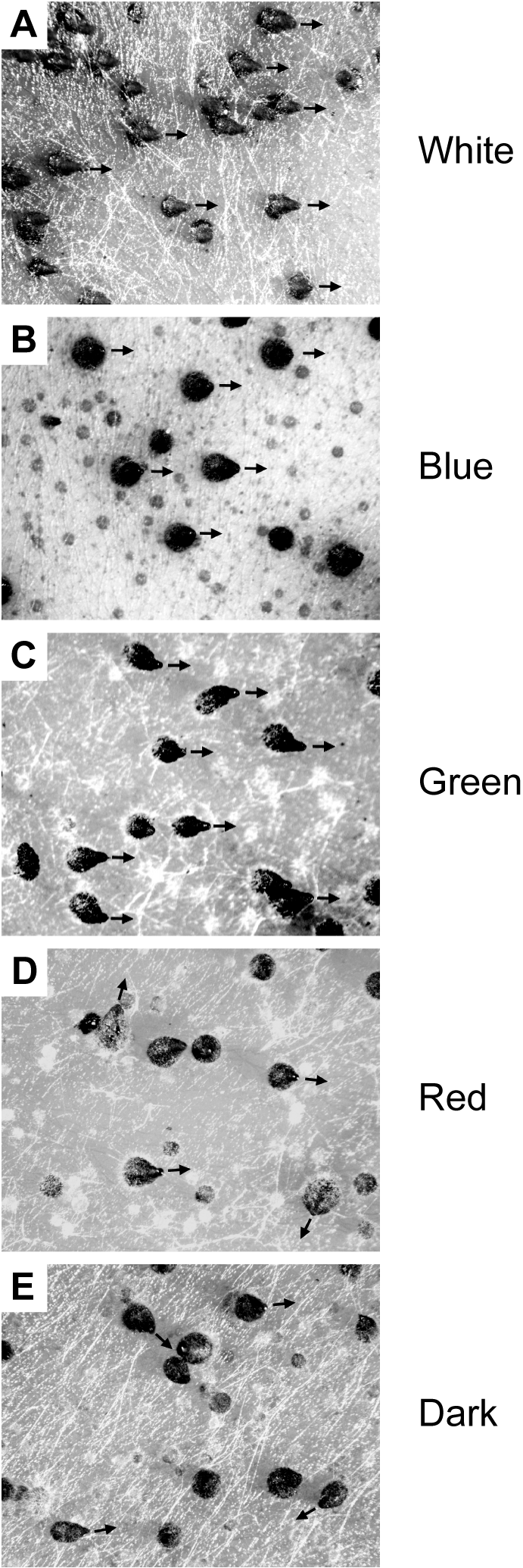

Supplement: Figure S8 — Perithecial neck phototropisms in response to unilateral light of different wavelengths. (A–C) Positive neck phototropisms in response to white light (A, fluence rate 3.4 µM/m2*s), blue light (B, wavelength: 470 nm; fluence rate 6.3 µM/m2*s), and green light (C, wavelength: 530 nm; fluence rate 3.4 µM/m2*s). (D, E) No neck phototropisms under red light (D, wavelength: 680 nm; fluence rate 5.4 µM/m2*s) or complete darkness (E). The arrows indicate the direction of neck tropisms. (1.08 MB TIF) [file pgen.1000891.s008.tif]

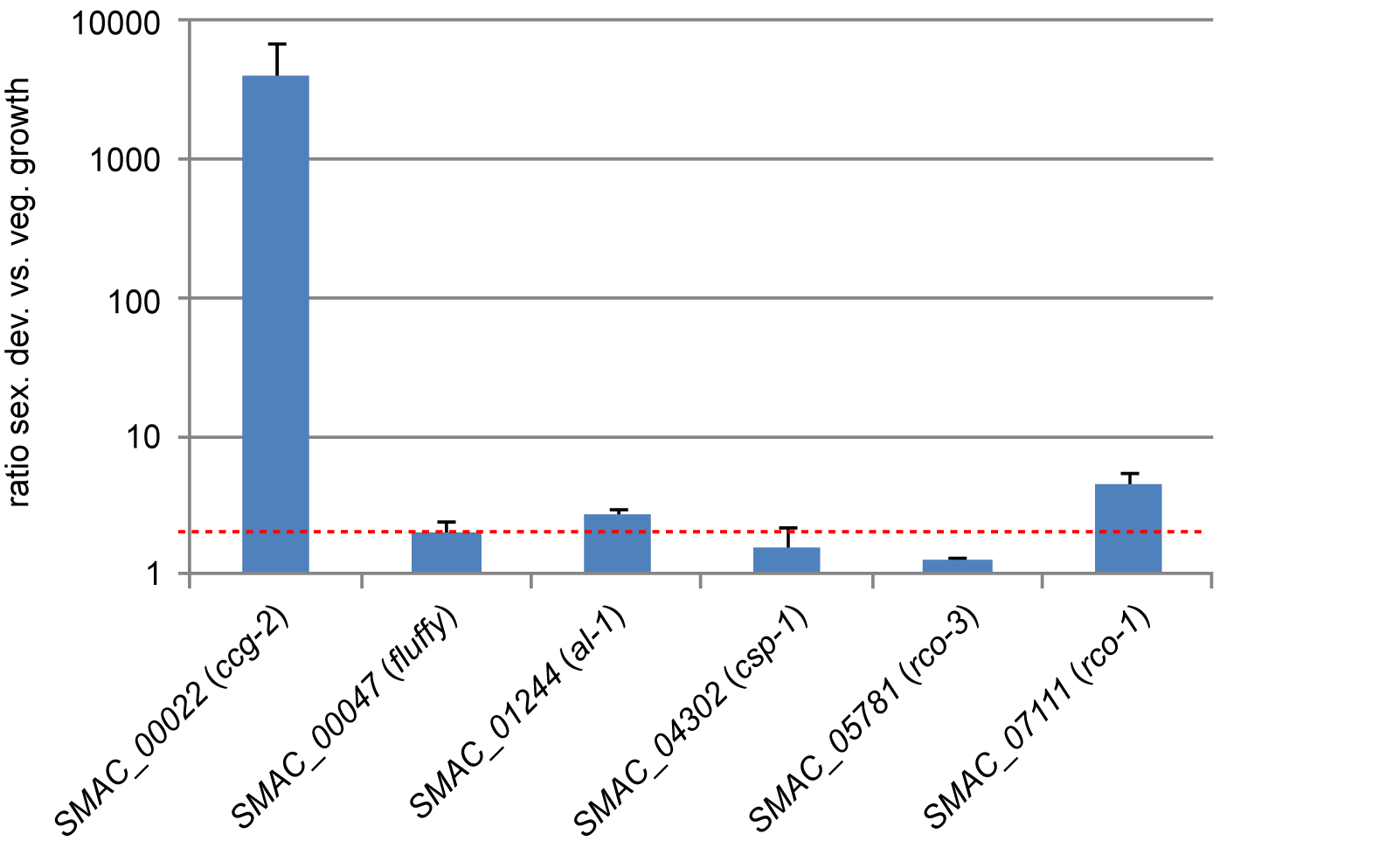

Supplement: Figure S9 — Expression of S. macrospora genes that are orthologs of genes involved in conidiation in N. crassa. Transcript levels were compared between sexual development and vegetative growth. Expression data are the results of two independent experiments and were determined by quantitative real time PCR. The red dashed line indicates two-fold upregulation. In N. crassa, the corresponding orthologs are regulators of conidiation (fluffy, csp-1, rco-1, rco-3) or encode structural proteins (ccg-2) or enzymes (al-1) that are important for conidiospore morphology. All six S. macrospora orthologs are transcribed both during vegetative growth and sexual development, and several are upregulated during sexual development. The strongest upregulation is observed in the ccg-2 ortholog SMAC_00022. In N. crassa, ccg-2 encodes a hydrophobin that forms the hydrophobic coat (rodlet layer) of the conidial cell wall (Bell-Pedersen et al. 1992 Genes Dev 6: 2382–2394). Generally, fungal hydrophobins are expressed when hyphae encounter an air/water interface (Wösten 1991 Annu Rev Microbiol 2001. 55:625–646), and this might be the reason why the S. macrospora ccg-2 ortholog is only weakly expressed in the submerged culture used to obtain vegetative mycelium but strongly upregulated under conditions for sexual development, i.e. during growth as a surface culture. The function of these genes in the aconidial S. macrospora is unknown. Several of the N. crassa orthologs have functions outside of conidiation, e.g. the putative transcriptional repressor rco-1 (Yamashiro et al. 1996 Mol Cell Biol 16: 6218–6228) or the glucose transporter rco-3 (Madi et al. 1997 Genetics 146: 499–508), but others like the transcription factor-encoding genes fluffy (Bailey and Ebbole 1998 Genetics 148: 1813–1820) and csp-1 (Lambreghts et al. 2009 Genetics 181: 767–781) are specific to conidiation and their function in S. macrospora remains to be elucidated. (0.21 MB TIF) [file pgen.1000891.s009.tif]

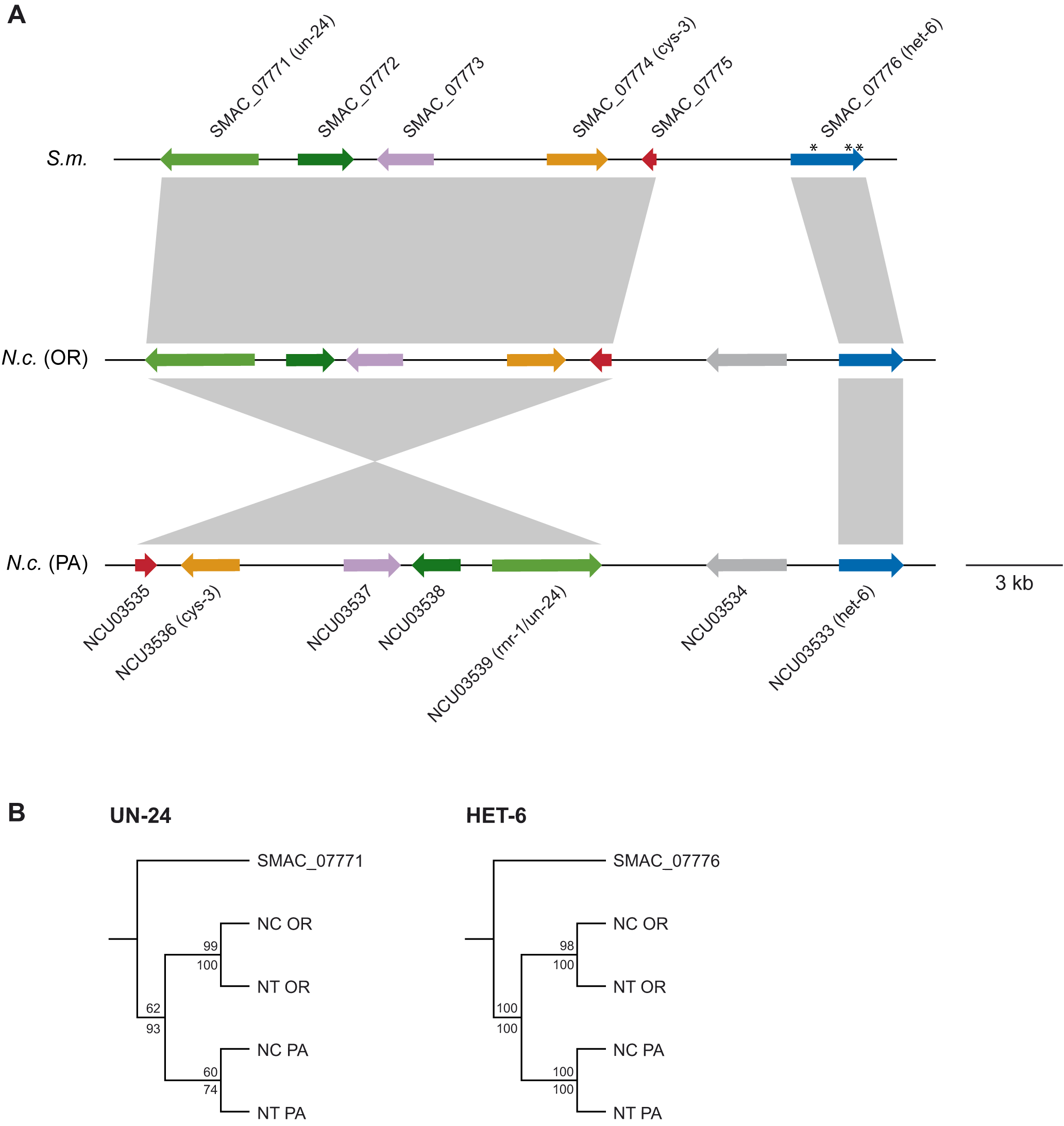

Supplement: Figure S10 — The het-6/un-24 locus from S. macrospora is syntenic to the OR allele of N. crassa. (A) Region from S. macrospora scaffold 5 and N. crassa scaffold 8 containing het-6 and un-24 genes. Homologous genes are given in the same color. The two different allelic combinations of het-6 and un-24 in N. crassa, Oak Ridge (OR) and Panama (PA), are indicated. The S. macrospora gene order resembles that of the Oak Ridge strain. SMAC_07776 contains three stop codons within the open reading frame (indicated by asterisks above the gene) and is therefore probably a pseudogene or it encodes a shorter HET-6. (B) Phylogenetic analysis of partial HET-6 and UN-24 proteins from S. macrospora, N. crassa (NC) and N. tetrasperma (NT). For N. tetrasperma, OR alleles were taken from strain P514, PA alleles from strain P2361 (Powell et al., Fungal Genet Biol 2007, 44: 896–904). The homologous P. anserina proteins were used as an outgroup to root the trees. Bootstrap values in % (10,000 bootstrap replicates) are given for maximum parismony and neighbor joining trees above and below the branches, respectively. OR and PA alleles from the two different Neurospora species cluster together whereas S. macrospora is basal to the Neurospora proteins indicating that the Oak Ridge gene order is probably ancient and the Panama gene order has arisen from an inversion after separation of the genus Neurospora from the genus Sordaria and before speciation of N. crasssa and N. tetrasperma. (0.18 MB TIF) [file pgen.1000891.s010.tif]

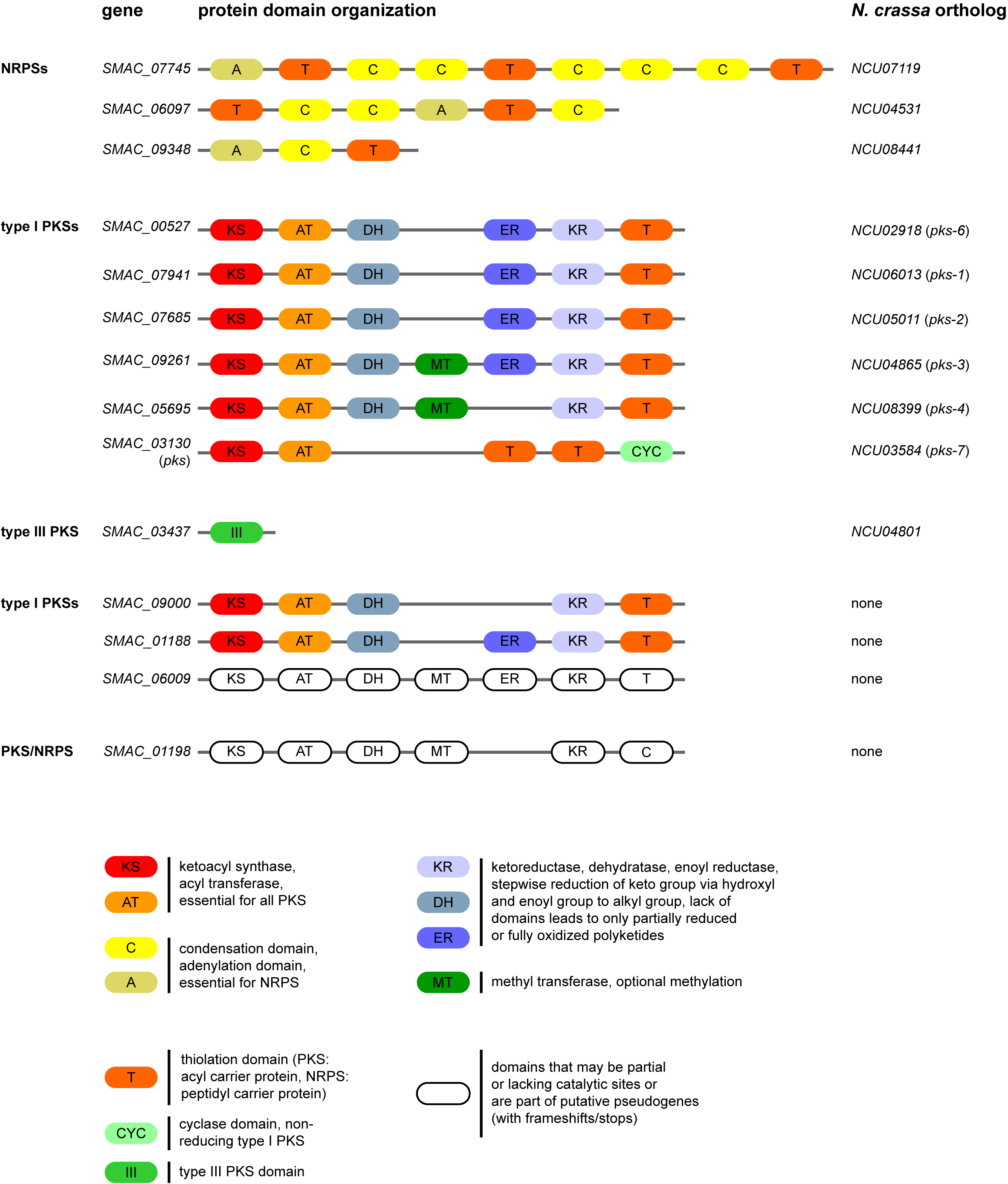

Supplement: Figure S11 — Summary of all proteins in S. macrospora that are predicted to be PKSs or NRPSs. (0.44 MB TIF) [file pgen.1000891.s011.tif]

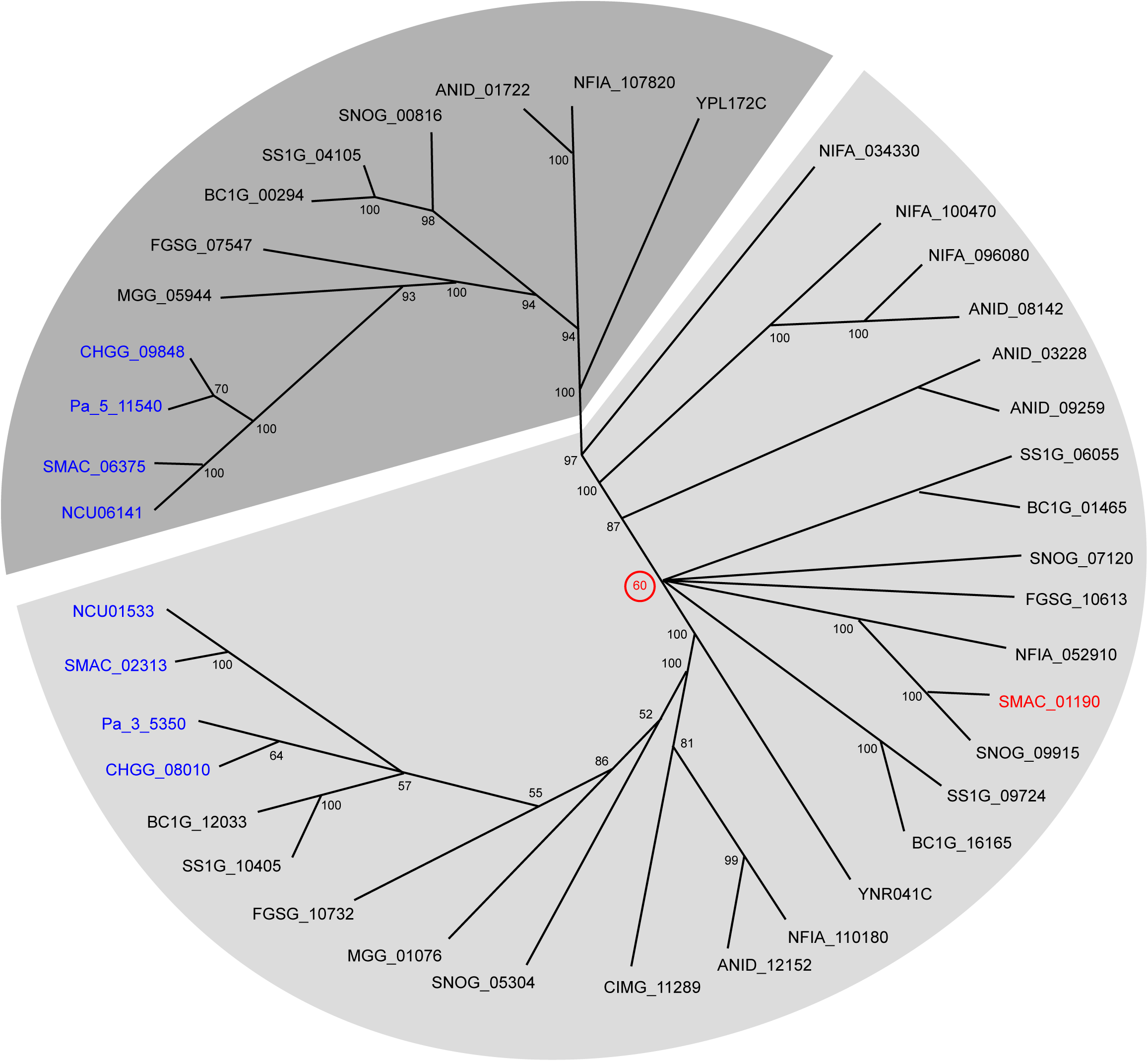

Supplement: Figure S12 — Phylogenetic analysis of the UbiA prenyltransferase family proteins from 12 fungi. The S. macrospora protein SMAC_01190 clusters in a group of “outsider” proteins with not quite clear phylogenetic resolution (bootstrap support of only 60 % as indicated in a red circle), most likely due to gene family expansion in Aspergillus/Neosartorya/Stagonospora/Botrytis/Sclerotinia. However, as it clusters with SNOG_09915, this might indicate horizontal gene transfer. Numbers at braches indicate bootstrap support (10,000 bootstrap replications) in % for the neighbor joining tree. Sordariales proteins are given in blue with the exception of the S. macrospora protein SMAC_01190 that clusters with the Dothideomycete Stagonospora nodorum and is given in red. Sequences that belong to the protoheme farnesyl transferase group are shaded in dark gray, sequences that belong to the polyprenyl transferase group are shaded in light gray. Sequences for P. anserina were obtained from the Podospora anserina genome project (http://podospora.igmors.u-psud.fr/index.html) and for all other fungi from the Fungal Genome Initiative of the Broad Institute at (http://www.broad.mit.edu/ annotation/fungi/fgi/index.html) or from our own data (S. macrospora). AN: Aspergillus nidulans, BC: Botrytis cinerea, CH: Chaetomium globosum, FG: Fusarium graminearum, MG: Magnaporthe grisea, NC: Neurospora crassa, NF: Neosartorya fischeri, PA: Podospora anserina, SM: Sordaria macrospora, SN: Stagonospora nodorum (Phaeosphaeria nodorum), SS: Sclerotinia sclerotiorum, Y: Saccharomyces cerevisiae. (0.33 MB TIF) [file pgen.1000891.s012.tif]
