# Supplementary material for: De novo Assembly of a 40 Mb Eukaryotic Genome from Short Sequence Reads: Sordaria macrospora, a Model Organism for Fungal Morphogenesis
Source: PLoS Genet. 2010 Apr 8;6(4):e1000891. doi: 10.1371/journal.pgen.1000891 (PMC2851567; doi:10.1371/journal.pgen.1000891)
Supplement: Table S1 — Assemblies with different coverage levels. (0.19 MB PDF) [file pgen.1000891.s013.pdf]

**Table S1.** Assembly with different coverage levels of short reads.

Assemblies were done with Velvet 0.7.56 with k=25. N50, maximum contig length (Nmax), the number of gaps that Velvet introduced within contigs and the total length of gaps within the assembly are given for different combinations of 454 coverage, coverage from one lane of Solexa single-end reads, coverage from a 300 bp Solexa paired-end library, and coverage from a 500 bp Solexa paired-end library. Addition of 454 reads has the most drastic effect on the number and length of gaps whereas addition of paired-end reads influences mostly N50 and Nmax (see Figure S2 for a graphical overview).

| solexa se cov. | solexa pe 300 bp library cov. | solexa pe 500 bp library cov. | 454 cov. | 454 pre-assembly included | N50 (kb) | Nmax (kb) | no. of gaps | total length of gaps (kb) | total length of assembly (Mb) | no. of contigs > 100 bp |
|---|---|---|---|---|---|---|---|---|---|---|
| 0 | 0 | 9 | 0 | no | 1 | 16 | 1999 | 630 | 1.1 | 2780 |
| 0 | 0 | 9 | 3 | no | 8 | 57 | 26866 | 4438 | 37.2 | 10038 |
| 0 | 0 | 9 | 6 | no | 33 | 148 | 12191 | 1791 | 38.9 | 5098 |
| 0 | 0 | 9 | 10 | no | 50 | 424 | 6311 | 877 | 39.1 | 5276 |
| 0 | 0 | 9 | 10 | yes | 72 | 409 | 4259 | 982 | 39.9 | 4810 |
| 0 | 0 | 18 | 0 | no | 1 | 24 | 51816 | 16593 | 24.5 | 33066 |
| 0 | 0 | 18 | 3 | no | 31 | 263 | 31073 | 4618 | 39.4 | 4184 |
| 0 | 0 | 18 | 6 | no | 81 | 394 | 10321 | 1401 | 39.0 | 3758 |
| 0 | 0 | 18 | 10 | no | 77 | 528 | 5464 | 707 | 39.1 | 4773 |
| 0 | 0 | 18 | 10 | yes | 121 | 688 | 3217 | 873 | 40.0 | 4540 |
| 0 | 0 | 27 | 0 | no | 3 | 47 | 147965 | 32801 | 50.8 | 30621 |
| 0 | 0 | 27 | 3 | no | 65 | 288 | 31931 | 3879 | 39.8 | 2995 |
| 0 | 0 | 27 | 6 | no | 92 | 526 | 9416 | 1183 | 39.1 | 3559 |
| 0 | 0 | 27 | 10 | no | 95 | 521 | 5070 | 6004 | 39.2 | 4141 |
| 0 | 0 | 27 | 10 | yes | 138 | 999 | 2781 | 742 | 40.0 | 4068 |
| 0 | 25 | 0 | 0 | no | 1 | 27 | 126427 | 21128 | 38.2 | 43909 |
| 0 | 25 | 0 | 3 | no | 8 | 51 | 24394 | 3878 | 39.7 | 10226 |
| 0 | 25 | 0 | 6 | no | 20 | 138 | 7163 | 1012 | 38.8 | 6144 |
| 0 | 25 | 0 | 10 | no | 25 | 187 | 3712 | 419 | 38.9 | 6179 |
| 0 | 25 | 0 | 10 | yes | 52 | 363 | 2048 | 648 | 39.7 | 4890 |
| 0 | 25 | 9 | 0 | no | 10 | 72 | 199453 | 25009 | 51.2 | 12071 |
| 0 | 25 | 9 | 3 | no | 53 | 356 | 27793 | 3724 | 40.5 | 3141 |
| 0 | 25 | 9 | 6 | no | 79 | 315 | 7713 | 998 | 39.1 | 3692 |
| 0 | 25 | 9 | 10 | no | 72 | 494 | 4247 | 481 | 39.1 | 4360 |
| 0 | 25 | 9 | 10 | yes | 112 | 995 | 2041 | 515 | 39.9 | 4218 |
| 0 | 25 | 18 | 0 | no | 39 | 177 | 183022 | 18024 | 49.5 | 3637 |
| 0 | 25 | 18 | 3 | no | 101 | 428 | 22893 | 2678 | 40.0 | 2589 |
| 0 | 25 | 18 | 6 | no | 98 | 395 | 6587 | 786 | 39.1 | 3542 |
| 0 | 25 | 18 | 10 | no | 90 | 432 | 4052 | 459 | 39.3 | 4219 |
| 0 | 25 | 18 | 10 | yes | 140 | 994 | 1759 | 356 | 39.7 | 4089 |
| 0 | 25 | 27 | 0 | no | 71 | 322 | 149008 | 13193 | 47.2 | 1942 |
| 0 | 25 | 27 | 3 | no | 110 | 529 | 18254 | 2002 | 39.6 | 2451 |
| 0 | 25 | 27 | 6 | no | 100 | 519 | 5912 | 681 | 39.1 | 3411 |
| 0 | 25 | 27 | 10 | no | 85 | 516 | 4143 | 448 | 39.3 | 4036 |

| | | | | | | | | | | |
|---|---|---|---|---|---|---|---|---|---|---|
| 0 | 25 | 27 | 10 | yes | 143 | 1060 | 1669 | 269 | 39.7 | 3975 |
| 0 | 50 | 0 | 0 | no | 16 | 96 | 151760 | 21276 | 54.9 | 7121 |
| 0 | 50 | 0 | 3 | no | 37 | 227 | 17721 | 2504 | 40.0 | 4070 |
| 0 | 50 | 0 | 6 | no | 42 | 257 | 4963 | 590 | 38.9 | 4882 |
| 0 | 50 | 0 | 10 | no | 32 | 189 | 3179 | 275 | 39.0 | 5698 |
| 0 | 50 | 0 | 10 | yes | 78 | 597 | 1164 | 211 | 39.6 | 4579 |
| 0 | 50 | 9 | 0 | no | 60 | 303 | 113488 | 13777 | 49.1 | 2529 |
| 0 | 50 | 9 | 3 | no | 94 | 407 | 13913 | 1806 | 39.6 | 2734 |
| 0 | 50 | 9 | 6 | no | 88 | 468 | 4781 | 585 | 39.1 | 3804 |
| 0 | 50 | 9 | 10 | no | 73 | 554 | 3530 | 369 | 39.3 | 4425 |
| 0 | 50 | 9 | 10 | yes | 117 | 727 | 1226 | 199 | 39.8 | 4296 |
| 0 | 50 | 18 | 0 | no | 87 | 563 | 81858 | 9052 | 45.4 | 1837 |
| 0 | 50 | 18 | 3 | no | 115 | 456 | 10740 | 1331 | 39.3 | 2628 |
| 0 | 50 | 18 | 6 | no | 98 | 565 | 4361 | 512 | 39.1 | 3657 |
| 0 | 50 | 18 | 10 | no | 90 | 417 | 3697 | 409 | 39.4 | 4244 |
| 0 | 50 | 18 | 10 | yes | 137 | 717 | 1251 | 211 | 39.8 | 4218 |
| 0 | 50 | 27 | 0 | no | 106 | 427 | 60715 | 6502 | 13.4 | 1692 |
| 0 | 50 | 27 | 3 | no | 120 | 568 | 8697 | 1047 | 39.2 | 2740 |
| 0 | 50 | 27 | 6 | no | 103 | 438 | 4265 | 509 | 39.3 | 3264 |
| 0 | 50 | 27 | 10 | no | 97 | 495 | 3860 | 440 | 39.6 | 4164 |
| 0 | 50 | 27 | 10 | yes | 137 | 992 | 1401 | 242 | 39.9 | 4128 |
| 9 | 0 | 9 | 0 | no | 1 | 19 | 32527 | 10586 | 19.8 | 46987 |
| 9 | 0 | 9 | 3 | no | 15 | 76 | 27046 | 3993 | 38.9 | 7094 |
| 9 | 0 | 9 | 6 | no | 45 | 251 | 9790 | 1381 | 38.9 | 4558 |
| 9 | 0 | 9 | 10 | no | 59 | 423 | 5233 | 664 | 39.0 | 4709 |
| 9 | 0 | 9 | 10 | yes | 88 | 492 | 3016 | 901 | 40.0 | 4399 |
| 9 | 0 | 18 | 0 | no | 3 | 24 | 135286 | 27896 | 48.4 | 36279 |
| 9 | 0 | 18 | 3 | no | 57 | 274 | 29463 | 3581 | 39.7 | 3325 |
| 9 | 0 | 18 | 6 | no | 86 | 511 | 8626 | 1168 | 39.1 | 3848 |
| 9 | 0 | 18 | 10 | no | 78 | 554 | 4669 | 602 | 39.3 | 5097 |
| 9 | 0 | 18 | 10 | yes | 137 | 713 | 2431 | 715 | 40.0 | 4757 |
| 9 | 0 | 27 | 0 | no | 124 | 917 | 180533 | 25537 | 50.9 | 13560 |
| 9 | 0 | 27 | 3 | no | 93 | 362 | 26715 | 2788 | 39.7 | 2477 |
| 9 | 0 | 27 | 6 | no | 96 | 782 | 7871 | 890 | 39.1 | 3445 |
| 9 | 0 | 27 | 10 | no | 89 | 761 | 4623 | 521 | 39.2 | 4154 |
| 9 | 0 | 27 | 10 | yes | 141 | 995 | 2192 | 628 | 40.0 | 4043 |
| 9 | 25 | 0 | 0 | no | 3 | 22 | 164032 | 24754 | 51.8 | 30025 |
| 9 | 25 | 0 | 3 | no | 13 | 67 | 22297 | 3358 | 40.1 | 7271 |
| 9 | 25 | 0 | 6 | no | 26 | 169 | 6032 | 788 | 38.8 | 5605 |
| 9 | 25 | 0 | 10 | no | 27 | 199 | 3181 | 331 | 38.9 | 6080 |
| 9 | 25 | 0 | 10 | yes | 60 | 326 | 1538 | 429 | 39.7 | 4768 |
| 9 | 25 | 9 | 0 | no | 22 | 120 | 164359 | 19256 | 51.0 | 6121 |
| 9 | 25 | 9 | 3 | no | 70 | 351 | 21012 | 2691 | 40.0 | 2891 |
| 9 | 25 | 9 | 6 | no | 83 | 353 | 6124 | 759 | 39.0 | 3713 |
| 9 | 25 | 9 | 10 | no | 68 | 443 | 3883 | 448 | 39.2 | 4407 |
| 9 | 25 | 9 | 10 | yes | 117 | 993 | 1581 | 387 | 39.8 | 4258 |

| | | | | | | | | | | |
|---|---|---|---|---|---|---|---|---|---|---|
| 9 | 25 | 18 | 0 | no | 62 | 297 | 131092 | 12927 | 47.2 | 2529 |
| 9 | 25 | 18 | 3 | no | 117 | 500 | 16351 | 1900 | 39.5 | 2643 |
| 9 | 25 | 18 | 6 | no | 105 | 456 | 5387 | 638 | 39.1 | 3625 |
| 9 | 25 | 18 | 10 | no | 92 | 598 | 3656 | 400 | 39.3 | 4277 |
| 9 | 25 | 18 | 10 | yes | 141 | 1023 | 1453 | 280 | 39.7 | 4140 |
| 9 | 25 | 27 | 0 | no | 97 | 418 | 101701 | 9393 | 45.0 | 1703 |
| 9 | 25 | 27 | 3 | no | 118 | 439 | 13041 | 1451 | 39.3 | 2596 |
| 9 | 25 | 27 | 6 | no | 104 | 593 | 4989 | 588 | 39.1 | 3514 |
| 9 | 25 | 27 | 10 | no | 87 | 611 | 3785 | 443 | 39.5 | 4148 |
| 9 | 25 | 27 | 10 | yes | 143 | 993 | 1480 | 253 | 39.8 | 4058 |
| 9 | 50 | 0 | 0 | no | 24 | 131 | 105216 | 14773 | 50.2 | 4875 |
| 9 | 50 | 0 | 3 | no | 44 | 223 | 12500 | 1746 | 39.4 | 3885 |
| 9 | 50 | 0 | 6 | no | 44 | 223 | 3954 | 456 | 38.9 | 4908 |
| 9 | 50 | 0 | 10 | no | 33 | 238 | 2793 | 235 | 39.0 | 5788 |
| 9 | 50 | 0 | 10 | yes | 86 | 597 | 937 | 198 | 39.6 | 4642 |
| 9 | 50 | 9 | 0 | no | 75 | 350 | 77601 | 9599 | 45.9 | 2164 |
| 9 | 50 | 9 | 3 | no | 95 | 392 | 10128 | 1317 | 39.3 | 2831 |
| 9 | 50 | 9 | 6 | no | 86 | 494 | 4085 | 469 | 39.1 | 3863 |
| 9 | 50 | 9 | 10 | no | 75 | 418 | 3241 | 342 | 39.4 | 4506 |
| 9 | 50 | 9 | 10 | yes | 121 | 1373 | 1061 | 180 | 39.7 | 4363 |
| 9 | 50 | 18 | 0 | no | 102 | 381 | 56525 | 6457 | 43.3 | 1748 |
| 9 | 50 | 18 | 3 | no | 109 | 403 | 8243 | 1008 | 39.1 | 2826 |
| 9 | 50 | 18 | 6 | no | 99 | 567 | 3989 | 451 | 39.2 | 3752 |
| 9 | 50 | 18 | 10 | no | 88 | 676 | 3469 | 394 | 39.5 | 4327 |
| 9 | 50 | 18 | 10 | yes | 137 | 803 | 1115 | 194 | 39.8 | 4290 |
| 9 | 50 | 27 | 0 | no | 123 | 372 | 42506 | 4712 | 41.9 | 1600 |
| 9 | 50 | 27 | 3 | no | 108 | 545 | 6964 | 825 | 39.1 | 2868 |
| 9 | 50 | 27 | 6 | no | 105 | 457 | 3915 | 464 | 39.3 | 3693 |
| 9 | 50 | 27 | 10 | no | 97 | 631 | 3778 | 452 | 39.7 | 4211 |
| 9 | 50 | 27 | 10 | yes | 140 | 992 | 1347 | 233 | 40.0 | 4184 |
