# Supplementary material for: De novo Assembly of a 40 Mb Eukaryotic Genome from Short Sequence Reads: Sordaria macrospora, a Model Organism for Fungal Morphogenesis
Source: PLoS Genet. 2010 Apr 8;6(4):e1000891. doi: 10.1371/journal.pgen.1000891 (PMC2851567; doi:10.1371/journal.pgen.1000891)
Supplement: Table S10 — Homologs to genes involved in light signaling and regulation. (0.05 MB PDF) [file pgen.1000891.s022.pdf]

Table S10. *S. macrospora* homologs of genes known or predicted to be involved in light sensing in *N. crassa*.

**locus_tag**	***N. crassa***		**function**
	**locus_tag**	**protein**	
SMAC_03527	NCU02356	WC-1	white collar-1, blue light receptor, transcription factor
SMAC_00185	NCU00902	WC-2	white collar-2, transcription factor, in complex with WC-1
SMAC_03705	NCU02265	FRQ	frequency, light signal transduction and circadian rhythmicity
SMAC_06136	NCU20238	VVD	blue-light sensing, PAS/LOV domain
SMAC_03470	NCU04834	PHY-1	phytochrome FphA, red/far-red sensing in *Aspergillus nidulans*
SMAC_07655	NCU05790	PHY-2	putative phytochrome, red/far-red sensing
SMAC_01274	NCU00582	CRY	putative blue-light sensing
SMAC_02423	NCU01731	VELVET	red- and blue-light sensing protein VeA in *Aspergillus nidulans*
SMAC_02424	NCU01735	ORP-1	opsin-related protein
SMAC_06025	NCU10055	NOP-1	homolog to bacteriorhodopsin
