# Supplementary material for: De novo Assembly of a 40 Mb Eukaryotic Genome from Short Sequence Reads: Sordaria macrospora, a Model Organism for Fungal Morphogenesis
Source: PLoS Genet. 2010 Apr 8;6(4):e1000891. doi: 10.1371/journal.pgen.1000891 (PMC2851567; doi:10.1371/journal.pgen.1000891)
Supplement: Table S11 — Homologs to genes involved in senescence. (0.07 MB PDF) [file pgen.1000891.s023.pdf]

Table S11. Identities and homologies of several age-related genes of *P. anserina* (*P.a.*) in *S. macrospora* (*S.m.*).

S. m.	***P. a.***	**UniProt acc. no.**	**function**	**id. [%][1]**	**e-value**
**protein quality control**					
SMAC_01730	*PaLon* Pa_3_4170	B2AZ54	ATP-dependent peptidase activity, mitochondrial protein quality	47	0.0
SMAC_2747	*PaClpP* Pa_2_3900	B2B591	ATP-dependent Clp protease, proteolytic subunit	64	3e-92
SMAC_04074	*PaIap* Pa_3_6030	B2B020	ATP-dependent peptidase activity, mitochondrial protein quality	70	0.0
SMAC_07050	*PaMap* Pa_2_5010	B2B5L3	ATP-dependent peptidase activity, mitochondrial protein quality	59	5e-68
**programmed cell death**					
----------	*PaAif1* Pa_1_22770	B2AS95	apoptosis-inducing factor 1	-----	-----
SMAC_09295	*PaAif2* Pa_4_460	B2AD94	apoptosis-inducing factor 2	78	0.0
SMAC_07439	*PaAmid1* Pa_5_4750	Q875G2	apoptosis-inducing factor (AIF)-like mitochondrion-associated inducer of death 1	70	e-167
----------	*PaAmid2* Pa_7_150	B2AP81	apoptosis-inducing factor (AIF)-like mitochondrion-associated inducer of death 2	-----	-----
SMAC_06729	*PaCypD* Pa_3_8900	B2AAR4	apoptosis regulation	62	3e-58
SMAC_08290	*PaMca1* Pa_5_5940	Q874X7	metacaspase, protein degradation	78	0.0
SMAC_00281	*PaMca2* Pa_1_690	B2A9D3	metacaspase, protein degradation	66	3e-158
SMAC_08654	*PaPrg3* Pa_5_4850	Q875F3	Aif-homolog	50	e-100
**DNA repair**					
SMAC_07943	*PaParp* Pa_0_940	B2ABS3	protein amino acid ADP-ribosylation	55	0.0
SMAC_08070	*PaUdg1* Pa_1_8860	B2AXU4	base-excision repair, uracil DNA N-glycosylase activity	65	4e-130
SMAC_06555	*PaNtg1* Pa_2_2170	B2B4R3	nuclear and mitochondrial base-excision repair	58	6e-147
**ROS scavenging**					
SMAC_05035	*PaSod1* Pa_1_17400	Q711T9	cytoplasmic copper/zinc antioxidant oxidoreductase	84	2e-77
SMAC_05700	*PaSod2* Pa_5_1740	B2AER6	cytoplasmic manganese antioxidant oxidoreductase	76	9e-108
SMAC_03915[2]	*PaSod3* Pa_2_4460	B2B5F1	mitochondrial manganese antioxidant oxidoreductase	76	8e-91
SMAC_05911	*PaPrx1* Pa_5_8240	B2AKR1	oxidoreductase activity, ROS-scavenging	61	2e-64
----------	*PaMth1* Pa_2_7880	Q9HGR1	SAM-dependent O-methyltransferase	-----	-----
**mitochondrial dynamics**					
SMAC_08468	*PaDnm1* Pa_1_12670	Q2WCN9	dynamin-related protein, mitochondrial fission	87	0.0
SMAC_01677	*PaFis1* Pa_3_3970	B2AZ34	mitochondrial fission	88	4e-82
SMAC_01873	*PaFzo1* Pa_7_6400	B2AW95	mitochondrial fission	69	0.0
SMAC_03955	*PaMgm1* Pa_2_2290	B2B4S5	dynamin-related protein, mitochondrial fusion	71	0.0
SMAC_04795	*PaMdv1* Pa_6_1780	B2B2X5	mitochondrial fission	77	0.0
**respiration**					
SMAC_08566	*PaAOX* Pa_3_1710	Q9C206	alternative oxidase, mitochondrial	69	2e-143
SMAC_05943	*PaCox17* Pa_1_8580	Q70J60	protein copper chaperone for respiratory chain complex IV	87	9e-26
**other**					
SMAC_03408	*grisea* Pa_4_8950	B2AR17	copper-regulated transcription factor	27	6e-31

[1]amino acid identity

[2]does not have a mitochondrial targeting sequence
