# Supplementary material for: De novo Assembly of a 40 Mb Eukaryotic Genome from Short Sequence Reads: Sordaria macrospora, a Model Organism for Fungal Morphogenesis
Source: PLoS Genet. 2010 Apr 8;6(4):e1000891. doi: 10.1371/journal.pgen.1000891 (PMC2851567; doi:10.1371/journal.pgen.1000891)
Supplement: Table S12 — Genes with putative functions in calcium signaling. (0.07 MB PDF) [file pgen.1000891.s024.pdf]

Table S12. *S. macrospora* homologs of genes involved in calcium signaling

locus_tag	N.c. ortholog	Best BlastP hit in non-redundant database		(putative) function
		acc. no.	organism[1]	
SMAC_00176	NCU09212	XP_959156		Ca^{2+} / CaM dependent protein kinase
SMAC_00194	NCU00916	CAE81985		Ca^{2+}/Na^{+} exchanger
SMAC_00614	NCU02826	XP_963783		Ca^{2+}/Na^{+} exchanger
SMAC_00692	NCU09871	XP_960153		Ca^{2+} and/or CaM binding protein
SMAC_00983	NCU06366	XP_962400		Ca^{2+}/H^{+} exchanger
SMAC_01000	NCU06347	XP_962381		Ca^{2+} and/or CaM binding protein
SMAC_01765	NCU04379	XP_957456		Ca^{2+} and/or CaM binding protein
SMAC_02320	NCU01564	XP_956201.1		Ca^{2+} dependent mitochondrial carrier protein
SMAC_02598	NCU03750	XP_961097		Ca^{2+} and/or CaM binding protein
SMAC_02648	NCU03804	Q05681		calcineurin A-1
SMAC_02674	NCU03833	P87072		calcineurin B-1
SMAC_03383	NCU04736	XP_960371.2		Calcium P-type ATPase
SMAC_03602	NCU08147	CAB65297		Calcium P-type ATPase
SMAC_03870	NCU07075	XP_960340		Ca^{2+}/H^{+} exchanger
SMAC_03941	NCU01241	XP_961607.1		mitochondrial carrier protein
SMAC_03962	NCU01266	CAE76127.1		phospholipase C
SMAC_04124	NCU05360	XP_963351		Ca^{2+}/H^{+} exchanger
SMAC_04252	NCU02762	XP_963732		Calcium channel subunit Cch1
SMAC_04280	NCU02738	XP_965123		Ca^{2+} binding protein PEF-1
SMAC_04466	NCU04120	XP_382067	*Gibberella zeae*	calmodulin
SMAC_04567	NCU03292	XP_964218		Calcium P-type ATPase
SMAC_04583	NCU03305	CAB65295		Calcium P-type ATPase
SMAC_04619	NCU09123	XP_958895		Ca^{2+} / CaM dependent protein kinase
SMAC_04743	NCU06617	XP_364625	*Magnaporthe grisea*	Ca^{2+} binding myosin regulatory light chain
SMAC_04807	NCU07711	XP_962297		Ca^{2+}/H^{+} exchanger
SMAC_04852	NCU09265	CAE76316		calreticulin
SMAC_05010	NCU02115	XP_963852		Ca^{2+} and/or CaM binding protein
SMAC_05273	NCU02175	XP_964245		phospholipase C
SMAC_05352	NCU06177	XP_962989		Ca^{2+} and/or CaM binding protein
SMAC_06270	NCU08490	XP_963419.1		Ca^{2+}/Na^{+} exchanger
SMAC_06559	NCU06650	XP_960883		phospholipase A2
SMAC_06632	NCU06703	XP_961018		Ca channel subunit Mid1
SMAC_07075	NCU06245	AAZ23805		phospholipase C
SMAC_07176	NCU05225	XP_961885		Ca-dep. mitochondrial NADH dehydrogenase
SMAC_07478	NCU06948	XP_958218		Ca^{2+} and/or CaM binding protein
SMAC_08401	NCU04898	XP_958407		cation-transporting ATPase 4
SMAC_08578	NCU07966	XP_962099		Cation-ATPase
SMAC_08748	NCU02283	XP_959927		Ca^{2+} / CaM dependent protein kinase
SMAC_08979	NCU05154	XP_956652		Calcium P-type ATPase

[1]organism name is given only if best hit is not a *N. crassa* protein
