# Supplementary material for: De novo Assembly of a 40 Mb Eukaryotic Genome from Short Sequence Reads: Sordaria macrospora, a Model Organism for Fungal Morphogenesis
Source: PLoS Genet. 2010 Apr 8;6(4):e1000891. doi: 10.1371/journal.pgen.1000891 (PMC2851567; doi:10.1371/journal.pgen.1000891)
Supplement: Table S13 — Genes with putative functions as motor proteins. (0.07 MB PDF) [file pgen.1000891.s025.pdf]

Table S13. *S. macrospora* genes with putative motor protein function

function	locus_tag	N.c. ortholog	BLASTP hit in non-redundant database	
			**acc. no.**	**protein name and organism**[1]
chitin synthase-myosin fusion protein	SMAC_01799	NCU04350	XP_956331	chitin synthase 6
Dynactin	SMAC_06671	NCU11177	XP_957728	
	SMAC_06897	NCU07196	XP_957421	dynactin subunit 5
	SMAC_07118	NCU04043	XP_957658	
	SMAC_07211	NCU03483	XP_955770	
	SMAC_07801	NCU03563	XP_956126	
	SMAC_07827	NCU04247	XP_961238	actin-2
Dynein heavy chain	SMAC_00761	NCU06976	XP_962616	dynein heavy chain
Dynein intermediate chain	SMAC_04637	NCU09142	XP_958914	
Dynein light chain	SMAC_06309	NCU03882	XP_956555	
	SMAC_05249	NCU09095	XP_964912	
	SMAC_05752	NCU02610	XP_001912639	*Podospora anserina*
Dynein light intermediate chain	SMAC_06004	NCU09982	XP_957965	
Kinesin	SMAC_00205	NCU00927	CAE76576	kinesin-related protein bimC
	SMAC_02573	NCU03715	XP_961491	
	SMAC_02750	NCU04581	XP_958282	
	SMAC_04212	NCU06832	XP_963673	
	SMAC_04781	NCU09730	XP_964432	kinesin heavy chain
	SMAC_05735	NCU02626	XP_964051	
	SMAC_06371	NCU06144	XP_960006	
	SMAC_06600	NCU06733	XP_960661	
	SMAC_07150	NCU05180	XP_961843	
	SMAC_07711	NCU05028	XP_956336	
myosin, class I	SMAC_05008	NCU02111	XP_964105	myosin-5
myosin, class II	SMAC_01243	NCU00551	XP_964712	
myosin, class V	SMAC_04508	NCU11354	CAD70976	
nud E	SMAC_08826	NCU08566	XP_962828	
nudF	SMAC_05575	NCU04312	Q7S7L4	Nucl. distrib. protein pac-1b
	SMAC_06094	NCU04534	Q7RY30	Nucl. distrib. protein pac-1a

[1]organism name is given only if best hit is not a *N. crassa* protein, protein name is given only if best hit is not a hypothetical protein
