# Supplementary material for: De novo Assembly of a 40 Mb Eukaryotic Genome from Short Sequence Reads: Sordaria macrospora, a Model Organism for Fungal Morphogenesis
Source: PLoS Genet. 2010 Apr 8;6(4):e1000891. doi: 10.1371/journal.pgen.1000891 (PMC2851567; doi:10.1371/journal.pgen.1000891)
Supplement: Table S14 — Genes with putative functions in MAP kinase and phospholipid signaling. (0.09 MB PDF) [file pgen.1000891.s026.pdf]

Table S14. *S. macrospora* genes with putative function in MAP kinase and phospholipid signaling.

**group**	**locus tag**	***N. crassa* ortholog**	**protein description**
Rho	SMAC_02216	NCU06454	CDC42
	SMAC_09071	NCU02160	RAC
	SMAC_06239	NCU01484	RHO1
	SMAC_03767	NCU08683	RHO2
	SMAC_01290	NCU00600	RHO3
	SMAC_08281	NCU03407	RHO4
RhoGEF	SMAC_07448	NCU06067	CDC24
	SMAC_01347	NCU00668	ROM1/2
	SMAC_05037	NCU02131	
	SMAC_04827	NCU10282	
	SMAC_04706	NCU06579	
	SMAC_08196	NCU09492	
	SMAC_004846	NCU02764	
RhoGAP	SMAC_04349	NCU02689	LRG1
	SMAC_01245	NCU00553	
	SMAC_00863	NCU07688	
	SMAC_05949	NCU02524	
	SMAC_06590	NCU09537	
	SMAC_08043	NCU10647	
	SMAC_02232	NCU00196	
	SMAC_00530	NCU02915	
	SMAC_07314	NCU07622	
	SMAC_07042	NCU01472	
RhoGDI	SMAC_04679	NCU06561	
RAS	SMAC_09275	NCU08823	RAS-1
	SMAC_03107	NCU03616	RAS-2
	SMAC_07014	NCU01444	similar to RheB
	SMAC_09077	NCU02167	KREV-1
	SMAC_06407	NCU06111	RAS-like
RasGEF	SMAC_05336	NCU06500	CDC25
	SMAC_08158	NCU09758	CDC25
	SMAC_02464	NCU01782	
	SMAC_08254	NCU03379	
RasGAP	SMAC_02368	NCU01642	
	SMAC_06277	NCU03852	
	SMAC_06397	NCU06122	
	SMAC_02847	NCU03116	IQGAP
PKA	SMAC_00682	NCU01361	kat SU
	SMAC_06240	NCU07080	kat SU
	SMAC_03233	NCU01166	reg SU
	SMAC_01638	NCU08377	CR1
MAPKKK	SMAC_05356	NCU06182	MIK2
	SMAC_02801	NCU03071	OS4
	SMAC_03673	NCU02234	MIK1
MAPKK	SMAC_06526	NCU04612	MEK2
	SMAC_01279	NCU00587	OS5
	SMAC_02183	NCU06419	MEK1
MAPK	SMAC_03492	NCU02393	MAK2
	SMAC_09127	NCU07024	OS2
	SMAC_05504	NCU11376	MAK1

PAK	SMAC_08843	NCU00406	CLA4
	SMAC_06321	NCU03894	STE20
Germinal center kinases	SMAC_01456	NCU00772	
	SMAC_04490	NCU04096	
	SMAC_04024	NCU01335	similar to SEPH
Phospho-inositide 3 kinase	SMAC_01337	NCU00656	VPS34
PI4 kinase	SMAC_01788	NCU04335	LSB6
	SMAC_04234	NCU09367	STT4
	SMAC_02169	NCU10397	PIK1
PI4P5 kinase	SMAC_08733	NCU02295	MSS4
PI3P5 kinase	SMAC_04978	NCU02083	FAB1
PI specific Phospholipase C	SMAC_03962	NCU01266	
	SMAC_07075	NCU06245	
	SMAC_05273	NCU02175	
	SMAC_07277	NCU11415	
PI phosphatase	SMAC_01583	NCU00896	SAC1
	SMAC_04020	NCU01330	Sca1-like
	SMAC_03774	NCU08689	FIG4
	SMAC_02638	NCU03792	
	SMAC_02726	NCU01047	
	SMAC_04575	NCU03298	synaptojanin-like
	SMAC_01362	NCU00684	
	SMAC_01920	NCU11185	myotubularin-like
	SMAC_00773	NCU06969	tensin-like
histidine kinase	SMAC_000616	NCU02815	
	SMAC_06529	NCU04615	
	SMAC_03740	NCU04834	
	SMAC_07655	NCU05790	
	SMAC_07859	NCU09520	
	SMAC_02513	NCU01833	
	SMAC_06862	NCU07221	
	SMAC_00215	NCU00939	
	SMAC_04951	NCU02057	
	SMAC_02891	NCU03164	
	SMAC_02506	NCU01823	
dynein heavy chain	SMAC_00761	NCU06976	
kinesin	SMAC_04781	NCU09730	Nkin
	SMAC_06600	NCU06733	
	SMAC_00205	NCU00927	
	SMAC_02573	NCU03715	
	SMAC_02750	NCU04581	
	SMAC_05735	NCU02626	
	SMAC_04212	NCU06832	
	SMAC_07711	NCU05028	
	SMAC_07150	NCU05180	
	SMAC_06371	NCU06144	
myosin	SMAC_04508	NCU11354	
	SMAC_05008	NCU02111	
	SMAC_01243	NCU00551	
	SMAC_01799	NCU04350	
