# Supplementary material for: De novo Assembly of a 40 Mb Eukaryotic Genome from Short Sequence Reads: Sordaria macrospora, a Model Organism for Fungal Morphogenesis
Source: PLoS Genet. 2010 Apr 8;6(4):e1000891. doi: 10.1371/journal.pgen.1000891 (PMC2851567; doi:10.1371/journal.pgen.1000891)
Supplement: Table S16 — S. macrospora homologs of conidiation-related genes from different ascomycetes. (0.07 MB PDF) [file pgen.1000891.s028.pdf]

Table S16. *S. macrospora* homologs of conidiation-related genes from different ascomycetes. Genes in this table are either involved in or differentially regulated during conidiation in *A. nidulans* or *N. crassa*.

locus tag	conidiation-related gene[1]	characteristics / remarks	e-value
SMAC_04005	*flbD*	Myb-DNA-binding protein	9e-30
SMAC_05750	*abaA*	regulatory protein AbaA	4e-11
SMAC_02716	*wetA*	regulatory protein WetA	4e-17
SMAC_04536	*con-6* (NCU08769)	conidiation-specific expression	2e-25
SMAC_02743	related to *con-6* (NCU01064)	related to conidiation protein CON-6	3e-26
SMAC_04887	*con-8* (NCU10977)	conidiation- specific expression	2e-23
SMAC_03018	*con-10* (NCU07325)	conidiation-specific expression	3e-36
SMAC_03017	*con-13* (NCU07324)	conidiation-specific expression	e-140
SMAC_01244	*al-1* (NCU00552)	phytoene dehydrogenase AL-1 (carotenoid biosynthesis, Conidiation- specific expression	0.0
SMAC_01277	*al-2* (NCU00585)	geranylgeranyl-diphosphate geranylgeranyltransferase, Conidiation- specific expression	0.0
SMAC_06570	*al-3* (NCU20300)	farnesyltranstransferase	0.0
SMAC_00047	*fl, fluffy, acon-1* (NCU08726)	C6-zinc finger TF, regulator of conidiation	0.0
SMAC_08071	*fr* (NCU07483)	frost, cell division control protein 1	0.0
SMAC_04302	*csp-1* (NCU02713))	conidial septation	e-152
SMAC_03705	*frq* (NCU02265)	frequency, clock protein FRQ	0.0
SMAC_06136	*vivid* (NCU20238)	blue light receptor	2e-98
SMAC_03527	*wc-1* (NCU02356)	zinc finger protein white collar-1, blue light receptor	0.0
SMAC_00185	*wc-2* (NCU00902)	zinc finger protein white collar-2	0.0
SMAC_02423	*ve-1* (NCU01731)	velvet, blue light receptor	0.0
SMAC_06025	*nop-1* (NCU10055)	opsin, green light receptor	e-143
SMAC_02601	*ccg-1, grg-1* (NCU03753)	GRG-1 glucose-repressible gene-1 protein	5e-33
SMAC_00022	*ccg-2, eas, bli-7* (NCU08457)	hydrophobin, rodlet protein	2e-023
SMAC_05970	*ccg-4* (NCU02500)	pheromone precursor, (clock-controlled gene-4)	e-135
SMAC_06579	*ccg-6* (NCU01418)	clock-controlled gene 6	5e-60
SMAC_05057	*ccg-8* (NCU09686)	clock-controlled gene-8	e-152
SMAC_05699	*ccg-9* (NCU10053)	trehalose synthase (clock-controlled gene 9)	0.0
SMAC_05106	*ccg-13* (NCU08907)	clock-controlled gene 13	3e-67
SMAC_05542	*ccg-15* (NCU08936)	clock-controlled gene 15, related to sporulation-specific gene SPS2	0.0
SMAC_00802	*bli-3* (NCU20927)	BLI-3 blue-light-inducible Bli-3 protein	e-116
SMAC_09428	*bli-4* (NCU08699)	BLI-4 putative oxidoreductase bli-4, mitochondrial	e-179
SMAC_07111	*rco-1* (NCU06205)	transcriptional repressor similar to *S. cerevisiae* TUP1	0.0
SMAC_04199	*rcm-1* (NCU06842)	regulator of conidia morphology, general transcriptional corepressor ssn6	0.0
SMAC_06671	*ro-2* (NCU111773)	ropy-2, dynactin arp1 p62 subunit RO-2	0.0
SMAC_04005	*rca-1* (NCU01312)	regulator of conidiation, Myb-like DNA-binding	e-179

		protein myb-1	
SMAC_05781	*rco-3* (NCU02582)	glucose transporter	0.0
SMAC_07195	*gna-3* (NCU05206)	G-protein alpha subunit	0.0
SMAC_06822	*rrg-1* (NCU01895)	response regulator	0.0
SMAC_00003	*hst-2* (NCU21250)	heat shock transcription factor	e-110
SMAC_04557	*cat-1* (NCU08791)	Catalase 1	0.0
SMAC_06468	*cat-3* (NCU00355)	Catalase 3	0.0
SMAC_05035	*sod-1* (NCU02133)	SOD-1 superoxide dismutase [Cu-Zn]	9e-83

[1]the first three genes (*flbD*, *abaA*, *wetA*) are from *A. nidulans*, all others from *N. crassa*. There are no homologs to the *A. nidulans* conidiation gene *brlA* present in *S. macrospora* or *N. crassa*, and no clear orthologs to the *A. nidulans* conidiation gene *flbC*.
