# Supplementary material for: De novo Assembly of a 40 Mb Eukaryotic Genome from Short Sequence Reads: Sordaria macrospora, a Model Organism for Fungal Morphogenesis
Source: PLoS Genet. 2010 Apr 8;6(4):e1000891. doi: 10.1371/journal.pgen.1000891 (PMC2851567; doi:10.1371/journal.pgen.1000891)
Supplement: Table S17 — S. macrospora homologs of genes involved in heterokaryon incompatibility from different ascomycetes. (0.06 MB PDF) [file pgen.1000891.s029.pdf]

Table S17. *S. macrospora* homologs of genes involved in heterokaryon incompatibility from different ascomycetes.

locus tag	species	gene	characteristics	e-value	reference
SMAC_05401	*N. crassa*	*mat A1* (NCU01958)	mating-type gene, transcription factor with alpha domain	e-126	(Glass et al., 1990)
SMAC_05404		*mat a1*	mating-type gene, transcription factor with HMG box	e-139	(Staben & Yanofsky, 1990)
SMAC_07218, SMAC_07220		*het-c* (NCU03493)	allelic *het* gene, signal peptide, transmembrane domain, glycin rich	0.0 0.0	(Saupe et al., 1996)
SMAC_07217, SMAC_07219		*pin-c* (NCU03494)	non-allelic *het* gene, TOL/HET-6/HET-E domain, interaction with *het-c*	0.0 0.0 0.0	(Kaneko et al., 2006)
SMAC_07776[1]		*het-6* (NCU03533), allele HET-6(PA)	allelic het gene, TOL/HET-6/HET-E domain	3e-102 1e-105	(Smith et al., 2000)
SMAC_07771		*un-24* (NCU03539)	allelic *het* gene, ribonucleoside-diphosphate reductase large chain	0.0	(Smith et al., 2000)
SMAC_08253		*tol* (NCU04453)	Suppressor of incompatibility TOL/HET-6/HET-E domain	6e-141	(Shiu & Glass, 1999)
SMAC_02578		*vib-1* (NCU03725)	Suppressor of incompatibility, nuclear localization domain	0.0	(Xiang & Glass, 2002)
SMAC_01205	*P. anserina*	*het-c2* AAA20542.1	non-allellic het gene against het-d and het-e, glycolipid transfer protein	2e-76	(Saupe et al., 1994)
SMAC_09206 SMAC_10006 SMAC_08962		*het-d* CAL30216.1	non-allelic *het* gene against *het-c*, WD repeat doamin, TOL/HET-6/HET-E domain, GTP-binding	5e-120 2e-79 2e-169	(Espagne et al., 2002)
SMAC_09206 SMAC_10006 SMAC_08962 SMAC_09419		*het-e* AAL37300	non-allelic *het* gene against *het-c*, WD repeat doamin, TOL/HET-6/HET-E domain, GTP-binding	9e-136 6e-134 0.0 2e-90	(Espagne et al., 2002)
SMAC_09206 SMAC_10006 SMAC_08962		*het-r* ACM48730.1	non-allelic *het* gene against *het-v*, WD repeat doamin, TOL/HET-6/HET-E domain, GTP-binding	3e-124 6e-91 0.0	(Chevanne et al., 2009)
SMAC_12582		*het-s* AAB8871	allelic *het* gene, prion analogue	2e-04	(Deleu et al., 1993)

[1]gene has internal stop codons
