# Supplementary material for: De novo Assembly of a 40 Mb Eukaryotic Genome from Short Sequence Reads: Sordaria macrospora, a Model Organism for Fungal Morphogenesis
Source: PLoS Genet. 2010 Apr 8;6(4):e1000891. doi: 10.1371/journal.pgen.1000891 (PMC2851567; doi:10.1371/journal.pgen.1000891)
Supplement: Table S18 — Predicted polyketide synthases (PKS), non-ribosomal peptide synthases (NRPS), and fatty acid synthases (FAS). (0.06 MB PDF) [file pgen.1000891.s030.pdf]

Table S18. Predicted polyketide synthases (PKS), non-ribosomal peptide synthases (NRPS) and fatty acid synthases (FAS). The *S. macrospora* genome contains three *nrps* genes, three *fas* genes, and seven *pks* genes all of which have a clear ortholog in *N. crassa* (first three parts of the table). In addition, *S. macrospora* has three additional *pks* genes and one *pks*/*nrps* hybrid gene for which no ortholog is present in *N. crassa* (last part of the table).

**locus_tag**	**Scaffold**	**N.c. ortholog**	**Best BLASTP hit in non-redundant database (NCBI)**		**enzyme**
			**acc. no.**	**protein name and organism**	
SMAC_07745	scaffold_65	NCU07119.2	ref|XP_960302.1	hypothetical protein NCU07119 [*Neurospora crassa* OR74A]	NRPS
SMAC_06097	scaffold_21	NCU04531.2	ref|XP_956942.2	hypothetical protein NCU04531 [*Neurospora crassa* OR74A]	NRPS
SMAC_09348	scaffold_138	NCU08441.2	emb|CAD70509.1	related to AM-toxin synthetase (AMT) [*Neurospora crassa*]	NRPS
SMAC_00841	scaffold_17	NCU07308.2	ref|XP_962466.1	fatty acid synthase alpha subunit reductase [*Neurospora crassa* OR74A]	FAS
SMAC_00840	scaffold_17	NCU07307.2	ref|XP_962465.1	fatty acid synthase beta subunit dehydratase [*Neurospora crassa* OR74A]	FAS
SMAC_00143	scaffold_7	NCU00056.2	ref|XP_955799.1	3-oxoacyl-[acyl-carrier-protein]-synthase [*Neurospora crassa* OR74A]	3-oxoacyl-[acyl-carrier-protein]-synthase
SMAC_03437	scaffold_2	NCU04801.2	pdb|3E1H_A	type III polyketide synthase [*Neurospora crassa*]	type III PKS
SMAC_03130	scaffold_20	NCU03584.2	emb|CAM35471.1	polyketide synthase [*Sordaria macrospora*]	type I PKS, non-reducing
SMAC_00527	scaffold_7	NCU02918.2	ref|XP_965600.1	hypothetical protein NCU02918 [*Neurospora crassa* OR74A]	type I PKS, reducing
SMAC_09261	scaffold_117	NCU04865.2	ref|XP_959122.1	hypothetical protein NCU04865 [*Neurospora crassa* OR74A]	type I PKS, reducing
SMAC_07685	scaffold_13	NCU05011.2	ref|XP_955938.1	hypothetical protein NCU05011 [*Neurospora crassa* OR74A]	type I PKS, reducing
SMAC_07941	scaffold_15	NCU06013.2	ref|XP_958135.2	hypothetical protein NCU06013 [*Neurospora crassa* OR74A]	type I PKS, reducing
SMAC_05695	scaffold_30	NCU08399.2	ref|XP_963238.1	hypothetical protein NCU08399 [*Neurospora crassa* OR74A]	type I PKS, reducing
SMAC_06009	scaffold_11	none	gb|AAR92212.1	polyketide synthase [*Gibberella moniliformis*]	type I PKS, reducing
SMAC_01198	scaffold_17	none	ref|XP_001274946.1	polyketide synthase, putative [*Aspergillus clavatus* NRRL 1]	hybrid PKS/NRPS
SMAC_01188	scaffold_17	none	ref|XP_001273596.1	polyketide synthase, putative [*Aspergillus clavatus* NRRL 1]	type I PKS, reducing
SMAC_09000	scaffold_106	none	ref|XP_001806097.1	hypothetical protein SNOG_15965 [*Phaeosphaeria nodorum* SN15]	type I PKS, reducing
