# Supplementary material for: De novo Assembly of a 40 Mb Eukaryotic Genome from Short Sequence Reads: Sordaria macrospora, a Model Organism for Fungal Morphogenesis
Source: PLoS Genet. 2010 Apr 8;6(4):e1000891. doi: 10.1371/journal.pgen.1000891 (PMC2851567; doi:10.1371/journal.pgen.1000891)
Supplement: Table S19 — A putative polyketide biosynthesis cluster that is partly conserved in S. macrospora and Phaeosphaeria nodorum. (0.06 MB PDF) [file pgen.1000891.s031.pdf]

Table S19. A putative polyketide biosynthesis cluster that is partly conserved in *S. macrospora* and *Phaeosphaeria nodorum*.

**locus_tag**	**closest homolog in *P. nodorum***	**predicted Pfam domains (see also Table S6)**	**Best BLASTP hit in non-redundant database (NCBI)**	**e-value**
SMAC_01188	SNOG_02561	PKS	ref|XP_001273596.1| polyketide synthase, putative [*Aspergillus clavatus* NRRL 1]	0.0
SMAC_01189	SNOG_05064		ref|XP_002148606.1| conserved hypothetical protein [*Penicillium marneffei* ATCC 18224]	2e-71
SMAC_01190	SNOG_09915	prenyltransferase	ref|XP_001800201.1| hypothetical protein SNOG_09915 [*Phaeosphaeria nodorum* SN15]	e-142
SMAC_01191	SNOG_09921	short chain dehydrogenase	ref|XP_001258624.1| short chain dehydrogenase (AtsC), putative [*Neosartorya fischeri*]	e-119
SMAC_01192	SNOG_08188	aldehyde reductase	gb|EEY20659.1| aldehyde reductase [*Verticillium albo-atrum* VaMs.102]	e-141
SMAC_01193	SNOG_09917		ref|XP_001800203.1| hypothetical protein SNOG_09917 [*Phaeosphaeria nodorum* SN15]	4e-55
SMAC_01194	SNOG_09918	FAD binding domain	ref|XP_001800204.1| hypothetical protein SNOG_09918 [*Phaeosphaeria nodorum* SN15]	e-178
SMAC_01195	SNOG_09916	Cytochrome P450	ref|XP_001800202.1| hypothetical protein SNOG_09916 [*Phaeosphaeria nodorum* SN15]	0.0
SMAC_01196	SNOG_09914	phenylalanine ammonia lyase	ref|XP_001800200.1| hypothetical protein SNOG_09914 [*Phaeosphaeria nodorum* SN15]	e-163
SMAC_01197	SNOG_09920	FAD binding domain	gb|EEY20661.1| FAD binding domain-containing protein [*Verticillium albo-atrum* VaMs.102]	0.0
SMAC_01198	SNOG_07866	PKS/NRPS hybrid, pseudogene	ref|XP_001274946.1| polyketide synthase, putative [*Aspergillus clavatus* NRRL 1]	0.0
SMAC_01201	SNOG_11395	Zn cluster transcription factor	ref|XP_001274942.1| hypothetical protein ACLA_055640 [*Aspergillus clavatus* NRRL 1]	6e-37
