# Supplementary material for: De novo Assembly of a 40 Mb Eukaryotic Genome from Short Sequence Reads: Sordaria macrospora, a Model Organism for Fungal Morphogenesis
Source: PLoS Genet. 2010 Apr 8;6(4):e1000891. doi: 10.1371/journal.pgen.1000891 (PMC2851567; doi:10.1371/journal.pgen.1000891)
Supplement: Text S1 — Parameters used for the assembly of the S. macrospora genome. (0.05 MB PDF) [file pgen.1000891.s032.pdf]

**Text S1.** Parameters used for the assembly of the *S. macrospora* genome.

**A.** Commands used for Velvet analysis. For the final assembly, Velvet 0.7.31 was run with the following parameters. Details on the input files can be found in Figure S1.

*./velveth velvet_out25contigs/ 25 -fasta -shortPaired s_1_fasta.txt s_2_fasta.txt s_3_fasta.txt s_4_fasta.txt -shortPaired2 s_6_fasta.txt s_7_fasta.txt s_8_fasta.txt -short sm1_single_fasta.txt 454_reads_clipped.txt -long Sorda_LargeContigs_oneLine.fasta*

*./velvetg velvet_out25contigs/ -exp_cov 80 -cov_cutoff 2 -min_contig_lgth 100 -ins_length 300 -ins_length2 500 -long_mult_cutoff 1*

**B.** Assembly and annotation of the rDNA unit

The rDNA unit (contig_4782) was assembled from 454 reads and contigs from the preassembled 454 reads. First, a BLASTN analysis of all 454 reads and the preassembled 454 contigs against the *N. crassa* and *M. grisea* full-length rDNA units as well as the *S. macrospora* ITS region was performed to identify reads with significant sequence identity to the rDNA region. These reads (1672 454 reads and 696 contigs) were then used for assembly of the rDNA unit with CodonCode Aligner version 3.0.3 (http://www.codoncode.com/aligner/). One of the resulting contigs contained the full-length rDNA unit (6488 bp). The 18S, 5.8S, and 26S rDNAs as well as the ITS regions 1 and 2 were annotated by comparison with the rDNA unit from *N. crassa* and the known ITS region from *S. macrospora*.

**C.** Assembly and annotation of the mitochondrial DNA

The mitochondrial DNA (scaffold_0) was assembled from 454 reads, contigs from the preassembled 454 reads, and contigs from the preassembled Solexa reads. First, a BLASTN analysis of all 454 reads, the preassembled 454 contigs, and the preassembled Solexa contigs against the *N. crassa* mitochondrial DNA was performed to identify reads with significant sequence identity to the mitochondrial DNA. These reads (5000 454 reads, 74 454 contigs, 85 Solexa contigs) were then used for assembly of the mitochondrial DNA with CodonCode Aligner version 3.0.3 (http://www.codoncode.com/aligner/). Ten of the resulting contigs covered most of the mitochondrial DNA and were manually to order them along the mitochondrial DNA of *N. crassa*, resulting in scaffold_0 (88432 bp, see also Table below) that constitutes the *S. macrospora* mitochondrial genome assembly. Annotation of CDSs, tRNAs, and rRNAs was done using ORF finder (http://www.ncbi.nlm.nih.gov/projects/gorf/) and manually annotating the results based on BLAST comparisons with the mitochondrial proteins, rRNAs, and tRNAs from *N. crassa* and *P. anserina*. For annotating the CDSs, the non-standard genetic code translation table 4 (http://www.ncbi.nlm.nih.gov/Taxonomy/Utils/wprintgc.cgi, for mold mitochondria) was used that translates TGA as Trp instead of stop. In total, 41 CDSs (12 of these within the large introns of other CDSs which encode mostly proteins from the respiratory chain), 23 tRNAs (including 2 tRNAs with anticodons for the TGA codon that is translated as Trp), and two rRNAs (LSU and SSU rRNA) were annotated (Figure S3, Table below).

Main features of the *S. macrospora* mitochondrial genome

| | |
|---|---|
| Size of the assembly | 88.4 kb |
| GC percentage (total genome) | 33.6 % |
| GC percentage in coding regions (CDSs) | 30.1 % |
| protein coding genes (CDSs) | 41 |
| tRNA genes | 23 |
| rRNA genes | 2 |
