# Supplementary material for: De novo Assembly of a 40 Mb Eukaryotic Genome from Short Sequence Reads: Sordaria macrospora, a Model Organism for Fungal Morphogenesis
Source: PLoS Genet. 2010 Apr 8;6(4):e1000891. doi: 10.1371/journal.pgen.1000891 (PMC2851567; doi:10.1371/journal.pgen.1000891)
Supplement: Text S2 — Predicted subcellular localizations, signal peptides, and transmembrane domains for the S. macrospora proteins. (0.08 MB PDF) [file pgen.1000891.s033.pdf]

Text S2. Predicted subcellular localizations, signal peptides, and transmembrane domains for the *S. macrospora* proteins. Localization was predicted with WoLFPSORT (Proceedings of the 4th Annual Asia Pacific Bioinformatics Conference APBC06, 2006, Taipei, Taiwan: pp. 39-48), signal peptides were predicted with SignalP 3.0 (J. Mol. Biol. 2004, 340: 783-795), transmembrane domains were predicted with HMMTOP (J. Mol. Biol. 1998, 283: 489-506) and TMHMM (J. Mol. Biol. 2001, 305: 567-580). S, signal peptide; A, signal anchor; Q, no signal peptide.

A. Localization and signal peptide predictions summary

**Localization**	**no. of peptides**	**S**	**A**	**Q**
cytoskeleton	264	3	0	261
cytosol	2370	78	32	2260
endoplasmic reticulum	8	3	2	3
extracellular	1239	986	59	194
Golgi apparatus	6	0	6	0
mitochondria	2112	330	63	1719
nuclear	3588	58	14	3516
peroxisome	25	0	0	25
plasma membrane	1177	197	241	739
total	10789	1655	417	8717

B. Summary of transmembrane domain (TM) predictions

no TM domains with both programs	6467
TM domains only with HMMTOP	2390[1]
TM domains only with TMHMM	28
at least one TM domain with both programs	1904

[1]of these, 437 are predicted to have a signal peptide with SignalP, this might be falsely predicted as TM domain.

C. Predictions of TM domains within putative plasma membrane proteins (as predicted by WolfPSORT)

no. of TM domains	HMMTOP predictions	TMHMM predictions
0	29	73
≥ 1	1148	1104
